# ML-QSAR and Fragment Selectivity for the Identification of Fourth-Generation EGFR^L858R/T790M/C797S^ Inhibitors

**DOI:** 10.3390/ijms27219796

**Published:** 2026-10-31

**Authors:** Guo-Gang Tu

**Affiliations:** Jiangxi Province Key Laboratory of Drug Target Discovery and Validation, School of Pharmaceutical Science, Jiangxi Medical College, Nanchang University, Nanchang 330006, China; tugg110@163.com or tuguogang@ncu.edu.cn

**Keywords:** machine learning, QSAR, fragment-based drug discovery, fourth-generation EGFR inhibitors, MD simulation

## Abstract

This study aimed to establish an integrative workflow that couples QSAR modeling based on ML-QSAR, fragment-level selectivity analysis, large-library virtual screening, and MD simulations to identify fourth-generation inhibitors of the EGFR^L858R/T790M/C797S^ mutant. Independent XGBoost models were trained on curated ChEMBL bioactivity data for mutant and wild-type EGFR, yielding strong predictive accuracy (*R*^2^ = 0.74–0.81; *RMSE* = 0.53–0.56) together with interpretable SHAP feature attributions. Fragment analysis highlighted structural motifs that were subsequently used to screen the ZINC database, from which 22 compounds with selectivity scores above 1.9 were retained. Docking and MD analyses identified three hits with strong selectivity, favorable binding energies (−25.3 to −36.4 kJ/mol) and interaction patterns consistent with known EGFR^L858R/T790M/C797S^ inhibitors. The pipeline can be transferred to other targets and provides a scalable template for rational, next-generation drug exploratory in precision oncology.

## 1. Introduction

**Figure 1:**
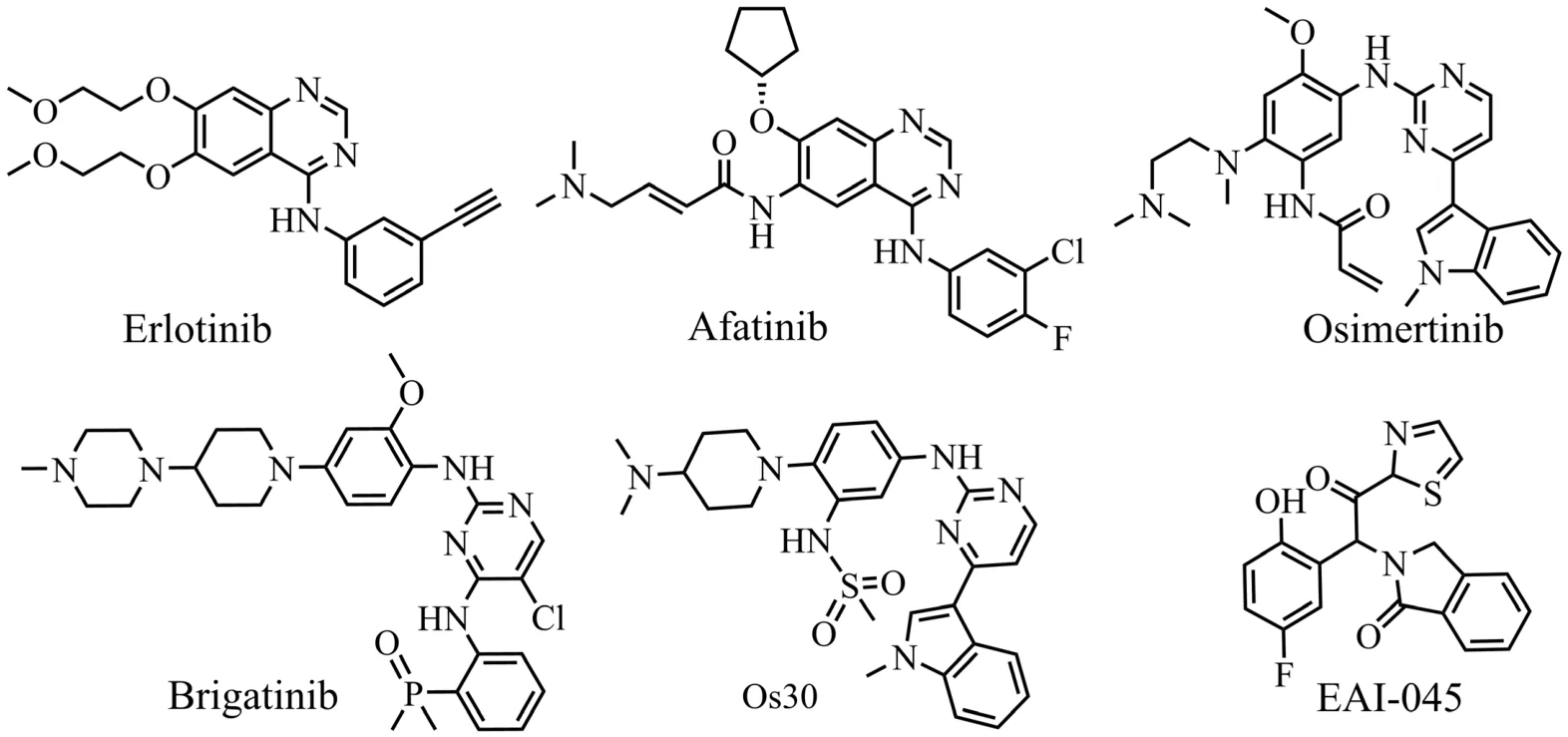
Chemical structures of representative EGFR inhibitors.

Lung cancer remains one of the most frequent and lethal malignancies worldwide, and non-small cell lung cancer (NSCLC) accounts for up to 80% of all cases [1]. Aberrant activation of the epidermal growth factor receptor (EGFR), through overexpression or activating mutation, drives tumorigenesis in a substantial subset of patients with NSCLC. Several small-molecule EGFR inhibitors have received US FDA approval for EGFR-mutant NSCLC (Figure 1). Erlotinib, a first-generation reversible inhibitor, was approved in 2004 for tumors harboring EGFR exon 19 deletions [2]; afatinib, a second-generation irreversible agent, later received approval for advanced or metastatic NSCLC carrying EGFR-activating mutations. Although it shares a Cys797-reactive acrylamide warhead with second-generation drugs, osimertinib is classified as a third-generation inhibitor because it preferentially suppresses EGFR^T790M^ resistance mutants while sparing the wild-type receptor [3]. Osimertinib entered clinical use in 2015 for EGFR^T790M^-positive NSCLC that had progressed on earlier therapies, and it was subsequently approved as a first-line treatment for metastatic NSCLC in 2018. Despite its substantial therapeutic benefit, acquired resistance ultimately limits the long-term effectiveness of osimertinib [4]. The most prominent resistance mechanism is the tertiary C797S mutation, which prevents the acrylamide group from forming a covalent bond with Cys797 [5]. Consequently, novel EGFR inhibitors that remain active against the C797S mutant are urgently needed [6].

Several investigational strategies have been proposed to overcome osimertinib resistance [7]. One mainstream approach exploits the Lys745 residue to design ATP-competitive inhibitors that remain effective against the EGFR^C797S^ mutant. Brigatinib, an FDA-approved ALK kinase inhibitor, was the first compound of this class; its oxazaphosphorinyl group engages the amino group on the Lys745 side chain and confers potent inhibition of EGFR bearing the tertiary C797S mutation. Because Lys745 is conserved within the αC-helix, however, this strategy carries a potential risk of off-target activity and raises safety concerns. Alternative approaches target the mutated Ser797 residue, as exemplified by compound Os30, or use allosteric inhibitors such as EAI-045, which have shown promising activity against the C797S mutant [8]. Despite these efforts, no inhibitor specifically designed to overcome the EGFR^C797S^ mutation has yet been approved [9].

Progress in machine learning and data-driven chemistry now makes it possible to combine heterogeneous bioactivity data and to resolve structure–activity relationships [10,11] that are difficult to capture with classical QSAR or purely structure-based approaches. In the present study, we assembled a computational pipeline that combines ML-QSAR modeling with fragment-based selectivity profiling and virtual screening to discover new fourth-generation EGFR inhibitors directed against C797S mutations. Specifically, the workflow was designed to (i) construct and validate predictive ML-QSAR models, (ii) identify the fragment motifs that govern selectivity, and (iii) screen candidate molecules predicted to combine high potency with strong selectivity toward EGFR^L858R/T790M/C797S^.

## 2. Results and Discussion

### 2.1. Dataset Analysis

After curation, two high-quality datasets were assembled: 6869 wild-type EGFR inhibitors and 881 mutant EGFR inhibitors. The wild-type set covered a pIC_50_ range of 4.0–11.2, while the mutant set ranged from 4.1 to 10.2, together spanning weak to highly potent compounds. Most compounds displayed moderate to high potency (pIC_50_ between 5.5 and 8.5), values that are typical of biologically relevant inhibitors and well suited to QSAR modeling [12].

Inspection of the Extended Connectivity Fingerprints (ECFP4) fingerprints showed that fewer than 6% of the 2048 bits were active per molecule, confirming the expected sparsity and structural diversity of circular fingerprints. The distribution of bit activation frequencies was strongly left-skewed due to most bits were rarely activated, and only a small subset displayed moderate activation. This profile indicates broad structural coverage with little feature redundancy in either dataset (Figure S1 in Supplementary Materials).

Butina clustering yielded many small- and medium-sized clusters, 65 clusters for the mutant set and 327 clusters for the wild-type set. The dominance of small clusters and few large clusters suggest high scaffold diversity and limited overrepresentation of particular chemotypes. The moderate pIC_50_ variance observed within clusters suggests that small structural changes can markedly alter potency, a feature that favors discriminative modeling (Figure S2 in Supplementary Materials).

Roughly 27% of the mutant inhibitors and 45% of the wild-type inhibitors fulfilled Lipinski’s Rule of Five [13], and the two groups displayed distinct physicochemical profiles, including molecular weight, calculated logP, hydrogen bond donor (HBD) and acceptor (HBA) counts, the QED score (Quantitative Estimate of Drug-likeness) [14] and the number of Lipinski violations (Figure S3 in Supplementary Materials). These profiles support the suitability of both datasets for selective drug discovery. Overlap analysis indicated that about 50% of the mutant compounds and 6% of the wild-type compounds also appeared in the other group, demonstrating limited structural similarity between the two collections (Figure S4 in Supplementary Materials).

### 2.2. Dataset Refinement and Clustering

The refinement protocol markedly reduced assay noise, outlier interference, and structural inconsistencies. Combining residual-based filtering and density-based spatial clustering of applications with noise (DBSCAN-based) structural screening [15] ensured that the retained molecules were both experimentally reliable and representative of their chemical neighborhoods. In total, 105 mutant and 736 wild-type compounds were flagged as anomalous. Venn analysis showed that the overlap between the two groups diminished after this step, implying that some anomalies arose from generic structural or experimental issues instead of group-specific chemistry (Figure S5 in Supplementary Materials). Removing these compounds improved dataset coherence and reduced the risk of bias in later modeling.

Clustering was repeated on the refined data with ECFP4 fingerprints followed by two-dimensional t-distributed stochastic neighbor embedding (t-SNE) projection, revealing more cohesive and chemically consistent groupings in both datasets (Figure S6 in Supplementary Materials). The number of clusters fell to 33 for the mutant set and 147 for the wild-type set. Importantly, the overall pIC_50_ distributions were essentially unchanged, confirming that refinement cleaned the data without shifting the bioactivity landscape.

### 2.3. Train–Test Sampling and Descriptors Selection

**Figure 2:**
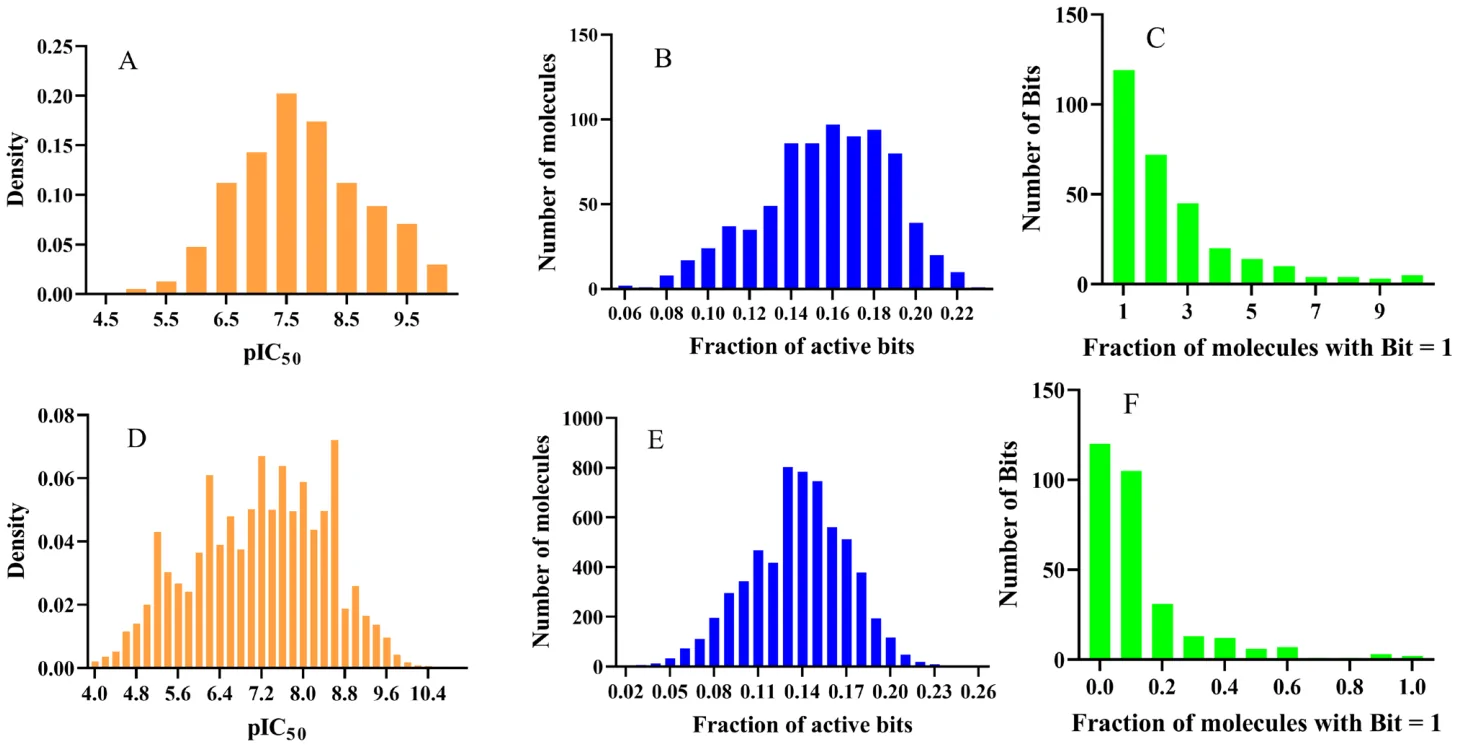
pIC_50_ distribution, ECFP4 sparsity, and fingerprint activation for EGFR inhibitors after descriptors selection. (**A**–**C**) mutant EGFR inhibitors, (**D**–**F**) wild-type EGFR inhibitors. Panels show (**A**,**D**) pIC_50_ distributions, (**B**,**E**) ECFP4 fingerprint sparsity and (**C**,**F**) fingerprint activation frequency, highlighting the substructural features retained after selection.

Cluster-stratified partitioning produced held-out test sets of 154 mutant compounds (19.8%) and 1230 wild-type compounds (20.1%) (Tables S1–S4 in Supplementary Materials). The test set should therefore be regarded as a cluster-aware held-out set for within-chemical-space evaluation rather than as a scaffold-disjoint external validation set. The ECFP4 fingerprints were highly sparse, with many bits rarely informative, consistent with substantial structural diversity. To improve interpretability and predictive power, descriptors were selected with Random Forest importance scores using the mean importance as the threshold [16]. After selection, pIC_50_ distributions, activation patterns and fingerprint sparsity were re-examined for each group (Figure 2). The final descriptor sets contained 296 bits for the mutant sets and 301 bits for the wild-type sets. In the selected fingerprints, 5–22% of bits remained active per molecule, and the distribution of activation-frequency became less left-skewed, reflecting reduced sparsity, lower redundancy, and clearer enrichment of the chemical patterns associated with bioactivity.

### 2.4. Machine Learning Modeling

Because the number of experimentally characterized EGFR^L858R/T790M/C797S^ inhibitors is limited, a triple-mutant-specific QSAR model could not be robustly trained. Therefore, a pooled mutant EGFR model was constructed using L858R, T790M, C797S, double mutants, and the triple mutant. Some inhibitors in this dataset are active against single-, double-, and triple-mutant EGFR, providing cross-mutant structure–activity information for candidate prioritization against the triple mutant. After hyperparameter tuning, both QSAR models showed improved stability and generalization [17]. The final hyperparameter settings are listed in Supplementary Table S5. On the train sets, *R*^2^ ranged from 0.74 to 0.80 and *RMSE* from 0.53 to 0.55, indicating a good fit. On the independent test sets, *R*^2^ values of 0.75–0.81 and *RMSE* values of 0.54–0.56 were obtained [18], confirming robust generalization and accurate pIC_50_ prediction. The *MAE* was also modest, ranged 0.40–0.42 on train data and 0.39–0.42 on test data. The complete performance metrics are summarized in Table 1, and the learning curves (Figure S7 in Supplementary Materials) showed near-synchronous convergence of train and validation *RMSE*, suggesting a limited bias-variance imbalance and effective capture of structure–activity trends. As comparation, a workflow of feature-selection using only train sets was repeated while retaining the same train/test split and XGBoost hyperparameters for mutant and wild-type EGFR inhibitors. Paired bootstrap analysis showed that these differences were small and also not statistically distinguishable (Figures S8 and S9 in Supplementary Materials).

### 2.5. Applicability Domain

**Figure 3:**
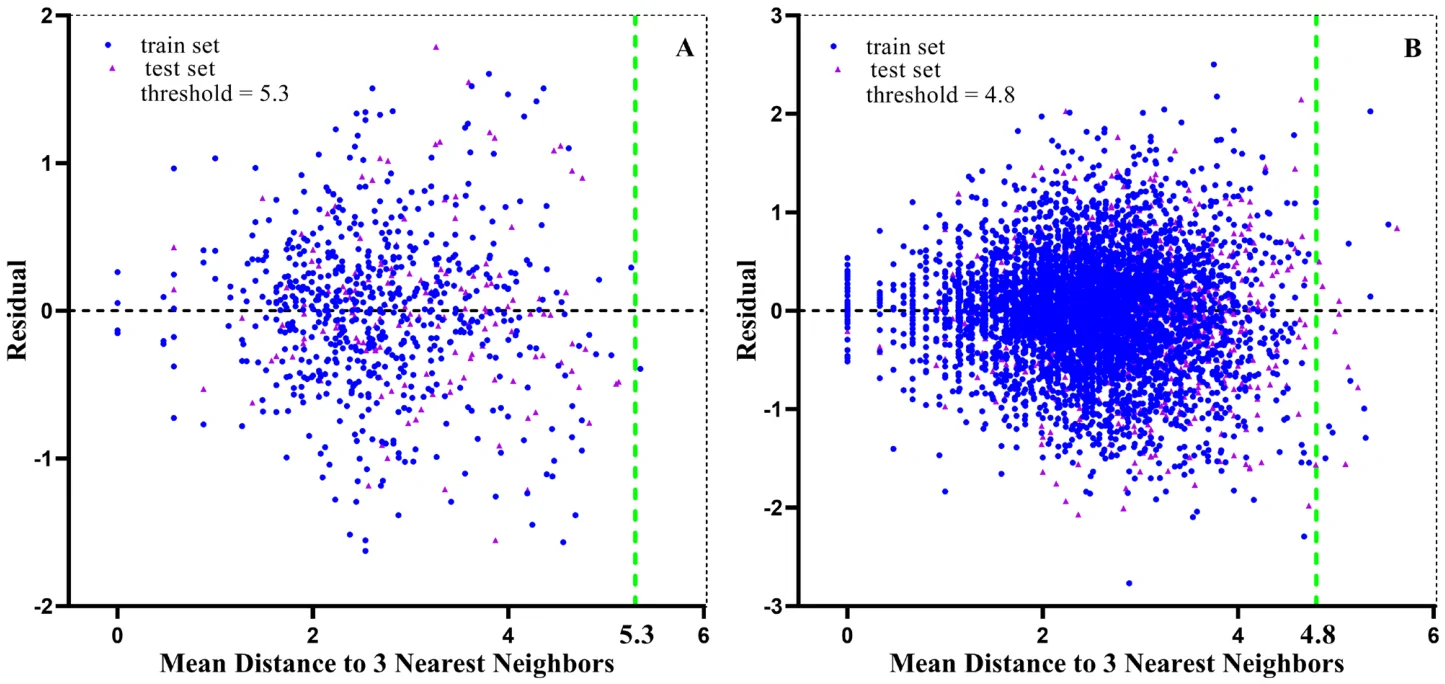
Applicability domain plots for the (**A**) mutant QSAR and (**B**) WT-QSAR models.

To gauge the robustness of the models, the chemical applicability domain (AD) was defined with a k-nearest-neighbors (kNN) approach [19]. For each query molecule, distances to its k nearest neighbors (*k* = 3) in the train set were calculated to provide a similarity measure relative to the modeled chemistry. A query was considered reliably predicted when the distance values fell within the defined threshold and the prediction residual was within ±3. A chemical with higher similarity, indicated by a larger number of close train neighbors, supported greater confidence. As shown in Figure 3, only one mutant compound (0.13%) and 19 wild-type compounds (0.31%) fell outside these criteria, confirming the reliability of both models.

### 2.6. Model Evaluation and Validation

**Figure 4:**
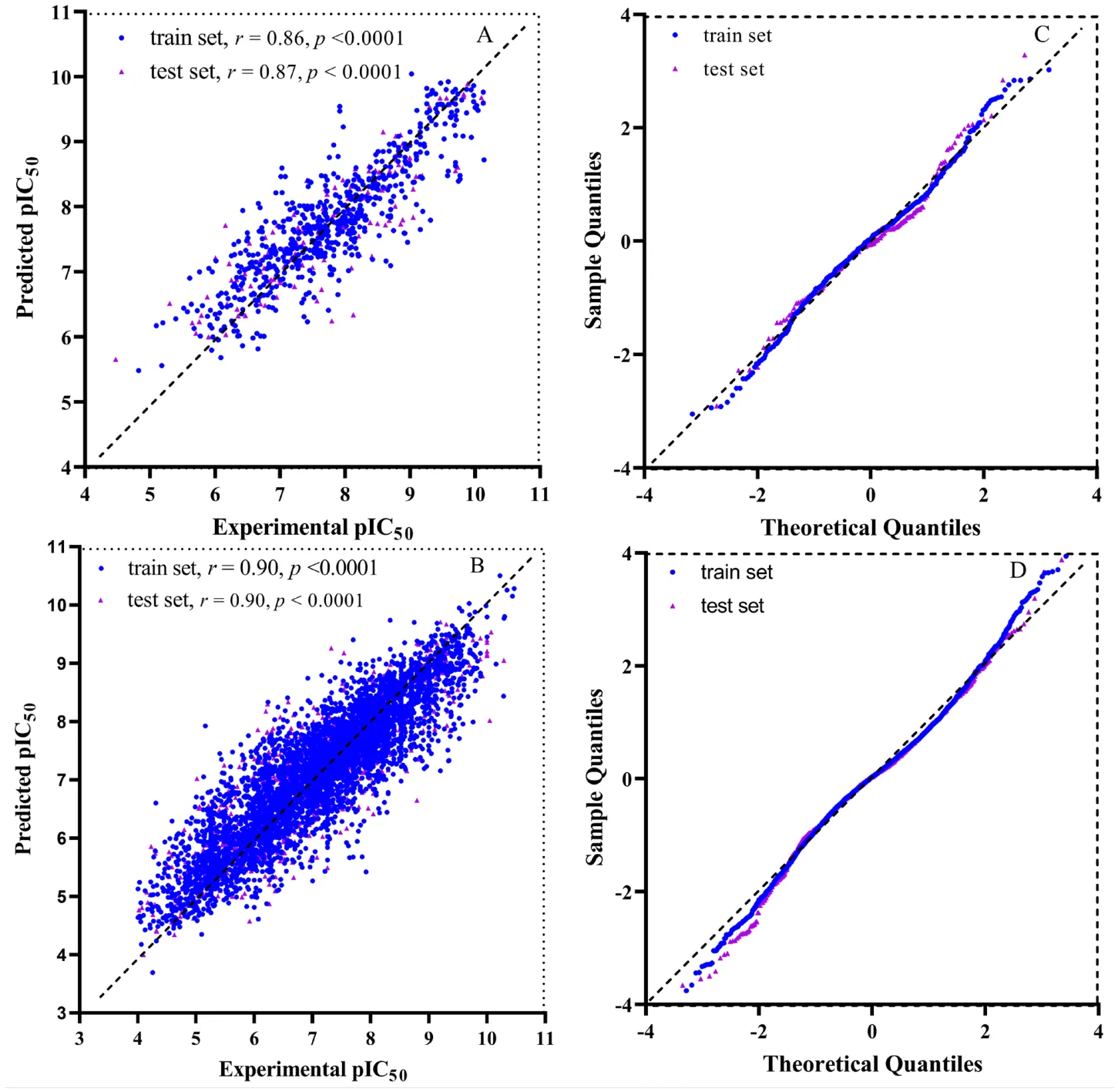
Overall performance of the XGBoost QSAR models. Panels show (**A**,**B**) experimental versus predicted pIC_50_ with Pearson correlation coefficients, and (**C**,**D**) residual Q-Q plots confirming normal error distributions for mutant-QSAR and WT-QSAR, respectively.

The Pearson correlation between predicted and experimental pIC_50_ values reached 0.86–0.90 for both models, indicating strong linear agreement and supporting the accuracy and stability of mutant-QSAR and WT-QSAR. Quantile–quantile (Q-Q) and residual analyses produced symmetric, zero-centered distributions with little systematic bias, consistent with good calibration (Figure 4). Together, these results indicate strong linearity, minimal systematic bias, and reliable calibration for both models.

Cross-dataset prediction further tested model specificity. The mutant-QSAR model was applied to the wild-type set and the WT-QSAR model to the mutant set. Each model performed well on its own data, but poorly on the opposite set with correlation coefficients of −0.55 and 0.42 for the two cross-prediction directions. Meanwhile some compounds (44.3%, 12.1%) fell outside the applicability domain (Figure S10 in Supplementary Materials). These results indicate that the models captured structural determinants specific to each group. A limitation of the present validation design is that the held-out test sets were generated by within-cluster sampling rather than by separating Bemis-Murcko scaffolds. Because no scaffold-disjoint external test set was used in this study, the present results should not be interpreted as evidence of generalization to unseen scaffolds; the predicted compounds remain subject to experimental validation.

### 2.7. SHAP-Based Interpretability

**Figure 5:**
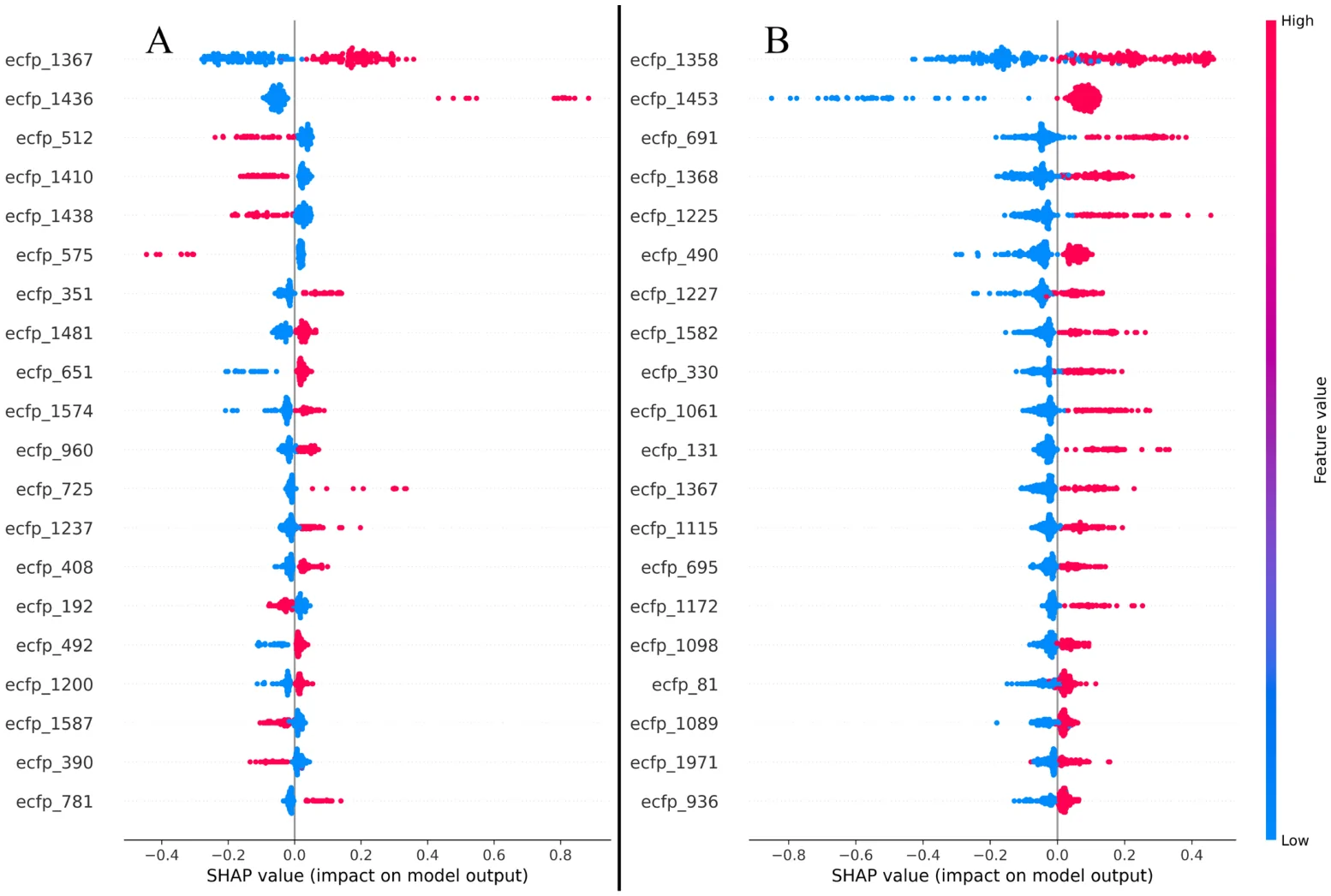
SHAP plots showing molecular feature contributions for the (**A**) mutant EGFR model and the (**B**) wild-type EGFR model. Feature labels denote the same ECFP4 bit indices in both panels; the rank order reflects model-specific feature importance.

SHAP analysis for models was used to reveal which molecular features drove individual predictions and thereby improve model interpretability. The SHAP explainer assigned a contribution value to every ECFP4 bit for the predicted pIC_50_ of each test compound across the cross-validation folds. Aggregating these values identified roughly 20 descriptors that consistently and strongly influenced each model output, pointing to substructural motifs important for selective EGFR inhibition. Representative SHAP summary plots are shown in Figure 5. Each plot ranks the fingerprint features by importance and indicates the direction of their influence on the predicted pIC_50_. In these plots, red indicates high feature values and blue indicates low feature values, while the *x*-axis spans positive and negative SHAP contributions to the predicted activity. The resulting distributions highlight distinct substructural determinants of selective inhibition and show that the models remain interpretable and consistent across validation folds. Global SHAP feature importance plots are provided in Supplementary Figure S11.

A trend was observed that molecules carrying more high-impact fingerprint bits tended to show progressively higher mean pIC_50_ values. Compounds containing all ten of the top SHAP-identified bits reached the highest observed and predicted activities, whereas molecules with fewer active bits showed a proportional decrease in potency. This monotonic trend is consistent with the view that key substructural features contribute additively to overall bioactivity [20] (Table S6 and Figure S12 in Supplementary Materials).

**Figure 6:**
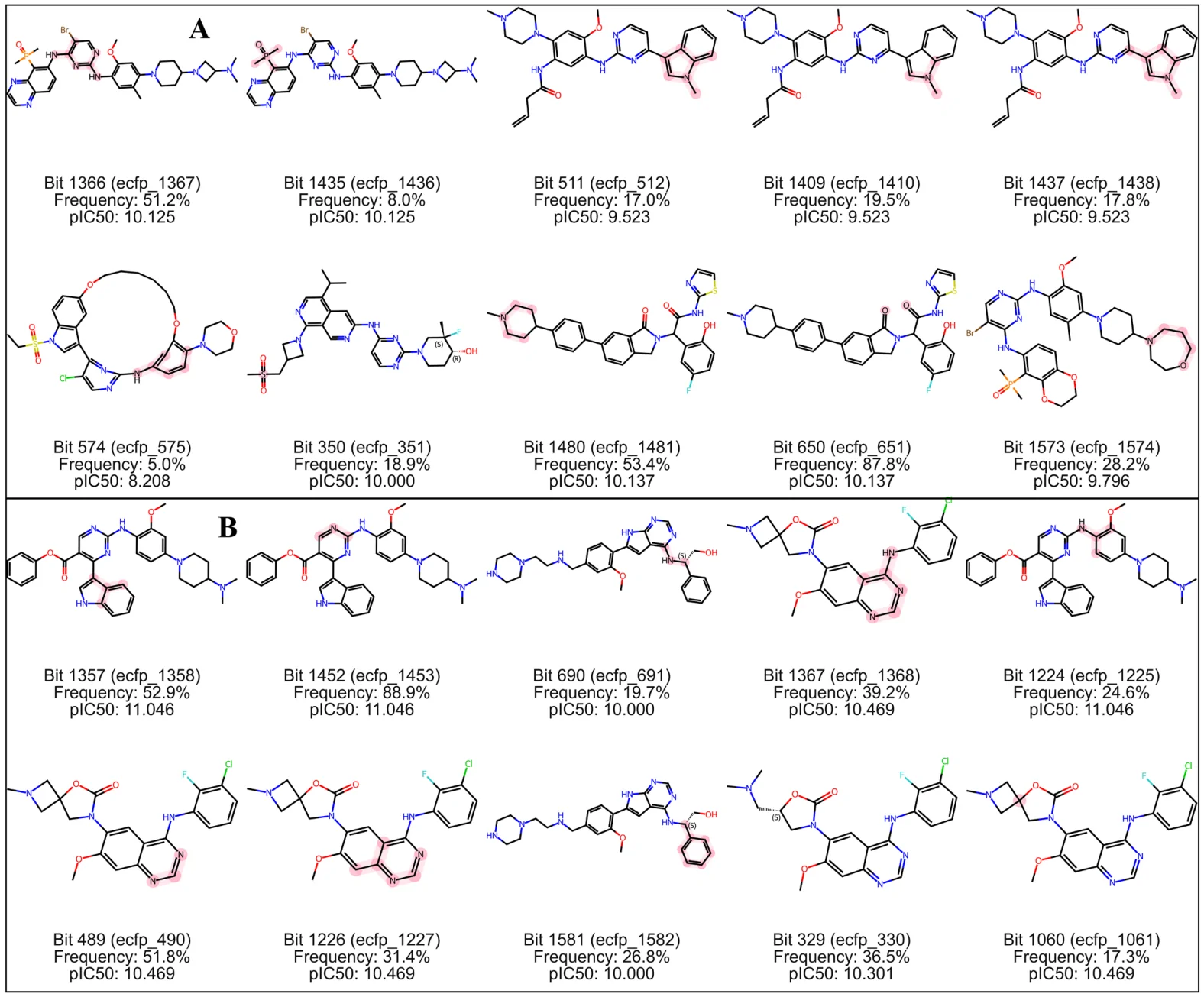
Visualization of the top 10 SHAP-identified fingerprint substructures mapped onto representative EGFR inhibitors, highlighting key substructures driving selective EGFR inhibition. (**A**): mutant inhibitors; (**B**): wild-type inhibitors. The displayed pIC_50_ value corresponds to the pIC_50_ of the representative molecule from which the highlighted fragment was derived.

The concordance of SHAP-based contributions with experimental activity trends suggests that the models captured authentic structure–activity relationships (SARs), not artifacts of descriptor encoding. To place the interpretation in a structural context, the ten most influential fingerprint bits were projected onto representative inhibitor structures (Figure 6). Fragment coloring marks high-impact bits, and when bit or hash collisions arise, the annotation reports the most common substructure and how frequently it occurs across the dataset, ensuring transparent interpretation of the feature-activity mapping.

### 2.8. Fragment-Based Virtual Screening

BRICS fragmentation of the EGFR inhibitor collection (6909 molecules) produced 2578 unique fragments. These fragments covered a wide chemical and physicochemical space with molecular weights from 13 to 1098, AlogP values from −2.83 to 9.03, hydrogen bond donors (HBD) from 0 to 6, hydrogen bond acceptors (HBA) from 0 to 10 and QED scores from 0.075 to 0.924 (Table S7 in Supplementary Materials). The broad ranges confirm a diverse fragment pool containing both larger hydrophobic and small polar scaffolds, well suited for fragment-based drug screening.

The mutant and wild-type models were then used to predict pIC_50_ values for the entire fragment library, after excluding fragments outside the applicability domain (Figure S13 in Supplementary Materials). Mean predicted values were 6.98 for the mutant set and 5.33 for the wild-type set. Selectivity scores against mutant EGFR ranged from 1.88 to 0.76, demonstrating broad diversity in fragment-specific activities (Table S8 in Supplementary Materials). The fragment with the highest selectivity score, SMILES [3*]ON[C@@H]([8*])CS(C)(=O)=O, was therefore selected for fragment-based virtual screening.

A similarity search of the drug-like ZINC collection was performed to retrieve molecules containing the selected fragment. The retrieved compounds were then scored with both models within the applicability domain. Molecules were retained when the selectivity score exceeded 1.9 and the predicted pIC_50_ against mutant EGFR was above 8.0. This filtering yielded 22 compounds for subsequent docking analysis (Table S9 in Supplementary Materials).

### 2.9. Docking and Molecular Dynamics Analysis

**Figure 7:**
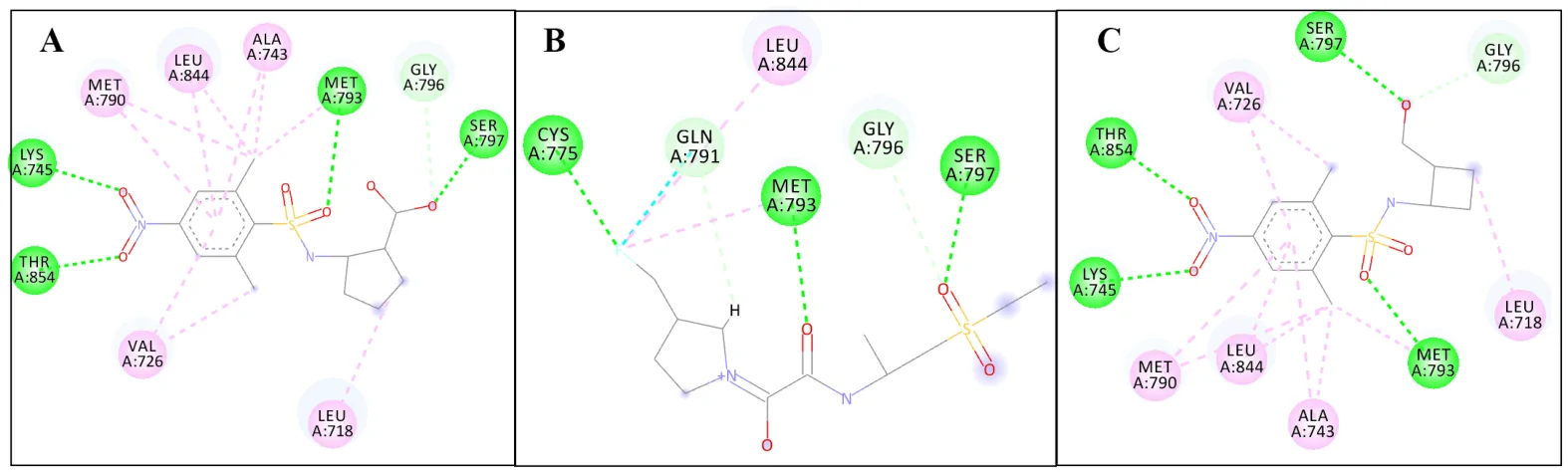
Docking analysis of (**A**) zinc563082375, (**B**) zinc1332126659 and (**C**) zinc1600303988 with the active site of EGFR^L858R/T790M/C797S^. Hydrogen bonds are showed by green dashed lines.

The QSAR predictions for the prioritized compounds were further computationally validated by the subsequent molecular docking and molecular dynamics simulations. Docking of the 22 selected compounds into EGFR^L858R/T790M/C797S^ (PDB ID: 8wd4) is shown in Figure 7 and Supplementary Figure S14. All compounds were able to occupy the active site of the mutant receptor, engaging in hydrophobic interactions and multiple hydrogen bonds with the protein. But only three compounds, zinc563082375, zinc1332126659 and zinc1600303988, formed hydrogen bonds with the mutant Ser797 residue, a feature considered critical for fourth-generation EGFR^L858R/T790M/C797S^ inhibitors. These three compounds were therefore advanced to molecular dynamics analysis.

**Figure 8:**
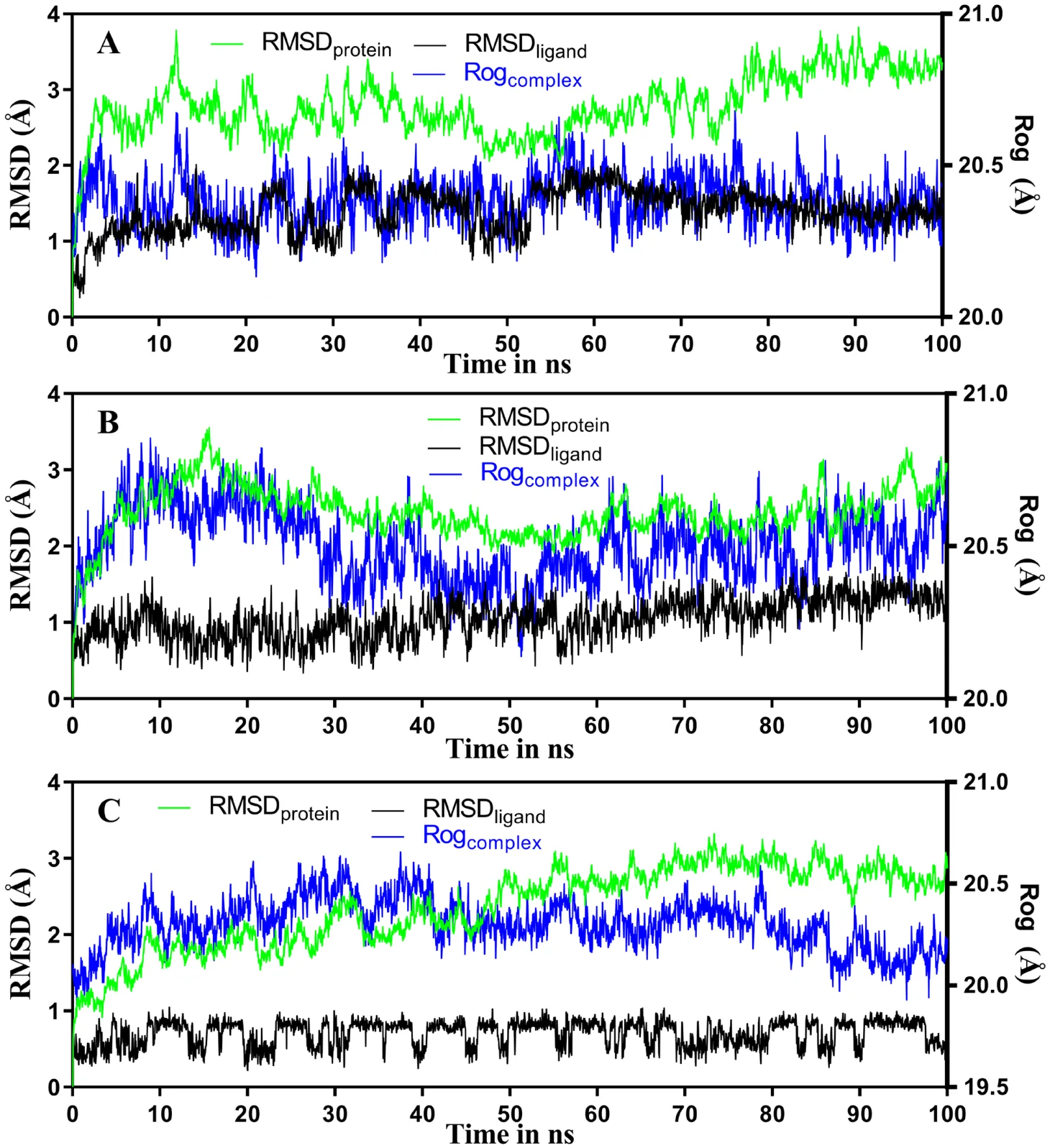
RMSD and RoG plots for the mutant EGFR-ligand complexes. (**A**): EGFR-zinc563082375, (**B**): EGFR-zinc1332126659, (**C**): EGFR-zinc1600303988.

**Figure 9:**
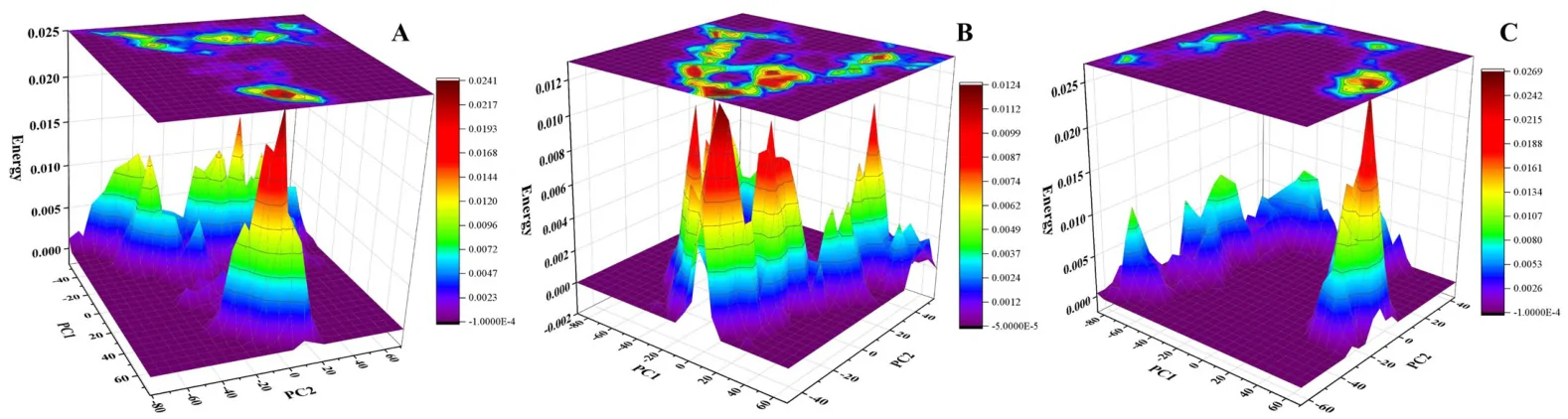
FEL plots for the mutant EGFR-ligand complexes. (**A**): EGFR-zinc563082375, (**B**): EGFR-zinc1332126659, (**C**): EGFR-zinc1600303988.

The three hit compounds were further evaluated by MD simulation. For comparison, 100 ns simulations were run for both the mutant EGFR-ligand and wild-type EGFR-ligand complexes. The resulting trajectories of RMSD, RoG, and FEL were inspected to judge dynamic stability. Figure 8 and Figure 9 present these profiles for the mutant complexes, while the corresponding wild-type plots are provided in Supplementary Figures S15 and S16. RMSD, a standard measure of structural deviation between conformations, indicated that all mutant complexes reached equilibrium after about 10 ns; the ligand and protein RMSD values remained below 2 Å and 4 Å, respectively, throughout the remainder of the simulation. These low values imply the absence of major conformational rearrangements and preservation of structural integrity. Complementary RoG analysis, which reflects three-dimensional compactness through the mass-weighted atomic distribution, showed fluctuations below 1 Å, indicating that the complexes did not undergo noticeable structural changes over the simulated period.

Free energy landscapes (FEL) were constructed from the first two principal components of each trajectory (Figure 9). In these plots, deep purple regions correspond to the lowest-energy conformers and deep red regions to the highest-energy conformers. The predominance of purple areas indicates that the sampled conformations are thermodynamically favorable and stabilize each complex during the simulation. Considered together, the RMSD, RoG and FEL results show that all of the protein–ligand complexes examined remained conformationally stable under the simulated conditions.

For fourth-generation EGFR^L858R/T790M/C797S^ inhibitors, hydrogen bonding with the mutant Ser797 residue is an important determinant of molecular recognition and binding affinity [21]. Table 2 summarizes the H-bond interaction efficiency of each hit compound with residues of mutant EGFR, listing the acceptor, donor, occupancy fraction (frac), the average distance (AvgDist) and average angle (AvgAng) for each interaction. All three mutant EGFR-ligand complexes formed stable hydrogen bonds with mutant Ser797, with occupancies of 59–77%. Additional hydrogen bonds with residues such as MET793 and ASP800 were also observed.

To characterize the molecular interactions of the hits inside the binding pockets of mutant and wild-type EGFR, binding free energies (Δ*G*) were calculated with the MM-PBSA approach [22]. The calculations decomposed Δ*G* into a van der Waals contribution from molecular mechanics, an electrostatic contribution to the solvation free energy obtained with the generalized Born model, and a nonpolar solvation contribution determined with an empirical model. In every complex, *E*_vdw_, *E*_ele_ and *E*_surf_ contributed favorably, reflecting attractive interactions between the ligand and the receptor, whereas *E*_GB_ was unfavorable, probably because of the low solubility of the complexes. All total binding free energies were negative, indicating that the complexes are stable, and the mutant EGFR complexes showed more favorable Δ*G*_binding_ values than their wild-type counterparts (Table 3). These energetic results agree with the XGBoost QSAR predictions and support the selective behavior of the EGFR^L858R/T790M/C797S^ inhibitors.

## 3. Materials and Methods

### 3.1. Dataset Acquisition and Curation

Bioactivity records for Homo sapiens EGFR inhibitors were collected from the ChEMBL database (target ID: CHEMBL203) [23]. Records with missing IC_50_ values or undefined activity units were discarded [24], and only entries annotated as Single Protein Format were kept to limit inter-assay heterogeneity. Measurements above 100,000 nM were treated as outliers and removed. Entries whose activity field contained comparison operators (>, <, ≤, ≥) rather than exact values were also removed. When the same compound had been tested in several assays, the median IC_50_ was adopted as the consensus potency. Potencies in nanomolar units were transformed to pIC_50_ using pIC_50_ = 9 − log_10_(IC_50_). Compounds were then partitioned according to the EGFR mutation status into a wild-type group and a mutant group, the latter covering L858R, T790M, C797S, L858R/T790M, T790M/C797S, L858R/C797S and L858R/T790M/C797S mutation. The two resulting sets were analyzed independently throughout the study to preserve data consistency.

### 3.2. Molecular Fingerprint Generation

The SMILES strings derived from each curated structure was converted into molecular descriptors with the ECFP4 algorithm [25]. Before fingerprint calculation, RDKit (version 2025.09.6) was used to standardize all molecules so that tautomers, protonation states and stereochemistry were represented uniformly. Fingerprints were then computed with radius of 2 and fixed length of 2048 bits to encode the substructural and topological information relevant to molecular recognition, while chirality were preserved to describe stereochemical and conformational features. The obtained binary vectors served as the input features for molecular clustering and for the subsequent machine-learning-based QSAR modeling.

### 3.3. Exploratory Dataset Analysis

Before model building, an exploratory analysis was carried out to inspect scaffold diversity, dataset quality, and structure–activity relationships. Because the bioactivity measurements came from different assay formats and experimental sources, both cheminformatics and statistical methods were combined to evaluate internal consistency and how representative the data were.

The range and uniformity of pIC_50_ values were inspected separately for the wild-type and mutant inhibitor sets. Bit activation and fingerprint sparsity frequencies were examined to gauge the information content and redundancy of the ECFP4, and frequency histograms of the active bits were also built to assess feature balance [26]. Molecular clustering on ECFP4 fingerprints was performed with the Butina algorithm using a Tanimoto similarity cutoff of 0.3 [27], which allowed structural diversity and within-group activity variation to be assessed for both datasets. The high-dimensional fingerprints were subsequently embedded in two dimensions by t-SNE to visualize cluster organization. Cluster sizes, average activity per cluster, and the presence of underrepresented regions or potential outliers were then examined. Physicochemical properties were profiled with Lipinski’s Rule of Five [28], and the chemical overlap between the wild-type and mutant sets was quantified to determine whether shared structures could compromise model specificity and selectivity.

### 3.4. Refinement and Clustering Dataset

The curated sets were further refined to suppress assay noise, remove outliers, and correct structural inconsistencies [29]. Anomalous compounds were detected by two complementary procedures, i.e., residual-based filtering and density-based clustering. In the first procedure, a Random Forest regressor configured with 100 estimators and random_state = 42 was subsequently trained on the ECFP4 fingerprints to reproduce experimental pIC_50_ values, and compounds whose absolute residuals ranked in the highest 5% (i.e., above the 95th percentile) were marked as activity outliers. In the second, DBSCAN [30], a density-based clustering algorithm for identifying noisy points, was applied to a Jaccard distance matrix computed from the binary fingerprints, with eps = 0.5 and min_samples = 4 chosen to balance sensitivity and cluster compactness. Molecules assigned the label −1 were regarded as structural outliers arising from rare or disconnected scaffolds. Any compound identified by either approach was removed before modeling to strengthen dataset robustness.

### 3.5. Train–Test Sampling and Descriptors Selection

Cluster-stratified partitioning was used to build the train and test sets [31]. Compounds were first grouped with Butina clustering [32] implemented in RDKit, using pairwise Tanimoto similarities calculated from the ECFP4 fingerprints at a cutoff of 0.3. Within each cluster, one-fifth of the molecules (20%) were randomly drawn into the test set, with the remaining 80% kept for train, ensuring that both sets covered similar scaffold space and that generalization could be assessed realistically. Feature selection was then carried out with a Random Forest regressor configured with 100 estimators and random_state = 42, which scored the importance of all 2048 fingerprint bits using scikit-learn. The NumPy (version 2.3.5) and Pandas (version 2.3.3) were used for data processing. Bits whose importance fell below the mean were discarded through a SelectFromModel procedure, leaving at most 500 informative descriptors. The refined train and test matrices, together with their pIC_50_ values, were passed to the regression stage.

### 3.6. Machine Learning Modeling

Separate predictive models, denoted mutant-QSAR and WT-QSAR, were built for the mutant and wild-type EGFR datasets with the XGBoost Regressor (XGBR) algorithm [33]. Ten-fold cross-validation was used for train and evaluation. In each fold, learning curves were generated by train on progressively larger portions of the train data, and these curves were inspected for signs of underfitting or overfitting and for convergence behavior. Cross-validation also served to average out noise caused by hash and bit collisions in the ECFP fingerprints, because repeated random partitions reduce the influence of any individual bit on the learned models.

Predictive accuracy and stability were quantified with four regression metrics, i.e., the coefficient of determination (*R*^2^), the root mean squared error (*RMSE*), the mean absolute error (*MAE*) and the mean squared error (*MSE*). For each metric, the mean value and its standard deviation computed over the ten-folds were recorded to assess consistency. Hyperparameters were optimized manually within the cross-validation procedure, with the aim of keeping model complexity balanced and avoiding overfitting.

To make the models interpretable, SHapley Additive exPlanations (SHAP) analysis was used to quantify the contribution of each fingerprint feature to the predictions [34]. SHAP values were calculated for all test compounds across the cross-validation folds and then aggregated into global summary plots and importance rankings. To examine how influential descriptors act together, compounds carrying different combinations of the ten most important SHAP-identified ECFP4 features were analyzed, and the corresponding mean predicted pIC_50_ values were plotted with error bars spanning the observed activity range. After optimization, the XGBR regressors were retrained on the complete train data to produce the final models used downstream. All modeling and visualization steps were implemented in Python (version 3.13.9) with the scikit-learn (version 1.9.0), XGBoost (version 3.2.0), SHAP (version 0.52.0), and Matplotlib libraries (version 3.10.6).

### 3.7. Model Evaluation and Validation

Each model was assessed on the independent test set reserved for its own group to gauge external predictive performance [35], with the Pearson correlation coefficient (*r*) between measured and predicted pIC_50_ values serving as the primary statistic. The residuals were screened for systematic bias using their distribution, quantile–quantile (Q-Q) plots, binned averages and per-bin root mean squared deviations (RMSD). To test cross-group transferability, the mutant and wild-type models were also applied to the opposite group’s data, and the resulting Pearson correlations were compared to determine how well each model discriminated between the two selectivity classes.

### 3.8. Fragment-Based Substructure Identification

All inhibitors in the curated datasets were dissected into chemically meaningful building blocks with the BRICS algorithm available in RDKit [36]. Because BRICS only cleaves synthetically accessible bonds, the resulting fragments can be systematically recombined and reused for activity analysis. Each fragment was encoded with ECFP4 fingerprints to capture its topological and substructural content, and the trained ML-QSAR models were then used to predict pIC_50_ values for every fragment against both mutant and wild-type EGFR. An empirical ranking score of fragment selectivity was expressed as the ratio of the mutant pIC_50_ to the wild-type pIC_50_ (Equation (1)).

(1) $$\mathrm{Selectivity}={\mathrm{pIC}}_{50}^{\mathrm{mutant}}/{\mathrm{pIC}}_{50}^{\mathrm{WT}}$$

For each fragment, drug-relevant physicochemical descriptors were computed, including the properties captured by Lipinski’s Rule of Five and the QED metric (Quantitative Estimate of Drug-likeness). Fragments combining favorable drug-likeness with strong selectivity were given priority in the subsequent virtual screening.

### 3.9. Similarity Search and Virtual Screening

Drug-like molecules for screening were obtained from the ZINC database [37], comprising approximately 981,247,974 compounds. Because this collection is far too large for direct docking or exhaustive prediction, a similarity search was applied as a first filtering step using the RDKit toolkit, which computed Tanimoto similarity between the selectivity fragment and the candidate structures on the basis of ECFP4 fingerprints (threshold = 0.1).

Once the predictive models were available, they were applied to estimate the mutant and wild-type EGFR inhibitory activity of the drug-like candidates, respectively. Only compounds lying within the models’ applicability domain were retained [38]. For each remaining molecule, a selectivity score was computed from the two predicted activities, and molecules combining high predicted mutant EGFR potency with a high selectivity score were selected as hits.

### 3.10. Molecular Docking and Dynamics Simulations

The crystallographic structures of wild-type EGFR (PDB ID: 1M17) [39] and mutant EGFR (EGFR^L858R/T790M/C797S^, PDB ID: 8wd4) [40] were obtained from the RCSB Protein Data Bank. Each hit compound was docked into the catalytic site of both receptors with AutoDock 4.2 [41], using the co-crystallized small-molecule ligands to define the binding region. A cubic search box of 50 × 50 × 50 Å with a point spacing of 0.375 Å was centered on the bound ligand coordinates with the default parameters for both receptors. Every docked complex (for PDB format in Supplementary Materials) was then submitted to 100 ns molecular dynamics simulation in the Amber package (version 22). Ligand parameters were assigned with the Leap module and Antechamber tools using the general AMBER force field (GAFF). Simulations employed the ff99SB protein force field, TIP3P water in an explicit cubic box leaving an 8 Å gap between the solute and the box walls, and chloride or sodium ions to neutralize the system. Long-range electrostatics were handled with the Particle Mesh Ewald (PME) method using a 12 Å cutoff, and bonds involving hydrogen atoms were constrained with the SHAKE algorithm. Prior to production runs, each system was minimized in two stages (i) water molecules and ions were relaxed over 2000 steps (1000 steepest descent followed by 1000 conjugate gradient) while the solute was held fixed with a 500 kcal/mol restraint, (ii) the entire system was minimized over 5000 steps (2500 steepest descent and 2500 conjugate gradient). The minimized complexes were warmed from 0 to 300 K while a weak 10 kcal/mol harmonic restraint kept the complexes fixed for 200 ps; this was followed by a 2 ns NVT equilibration at 300 K, and finally 100 ns unrestrained production simulations were run under NPT conditions at 300 K and 1 atm.

The stability of the protein–ligand complexes was evaluated with the root mean square deviation (RMSD) of the protein and ligands and the radius of gyration (Rog) [42]. Binding free energies were computed with the MM-PBSA.py tool of Amber. For every snapshot, the energy of the ligand, enzyme and complexes were calculated independently, and the overall Δ*G*_binding_ was obtained from Equation (2). In this framework, Δ*G*_gas_ denotes the gas-phase interaction energy, which includes van der Waals (*E*_vdw_), electrostatic (*E*_ele_), and internal strain terms (bond, angle, and dihedral energies). The solvation free energy (Δ*G*_sol_) was separated into nonpolar (*E*_surf_) and polar (*E*_GB_) contributions. The entropic term (TΔ*S*) was omitted, as computing it is computational cost and provides little predictive benefit.

Δ*G*_binding_ = Δ*G*_gas_ + Δ*G*_sol_ − TΔ*S* (2)

## 4. Conclusions

This study presents an integrated workflow that combines ML-QSAR modeling, fragment-level selectivity profiling, virtual screening of a large compound library, and molecular dynamics analysis to address the longstanding challenge of identifying fourth-generation EGFR^L858R/T790M/C797S^ inhibitors. Using curated mutant and wild-type EGFR inhibitors and interpretable ML-QSARs, the pipeline captured fine-grained determinants of selectivity and converted them into fragment-based screening criteria. The resulting hits combined strong predicted affinities with favorable drug-like properties and reproduced interaction patterns typical of known EGFR^L858R/T790M/C797S^ inhibitors, supporting the mechanistic plausibility of the identified chemotypes. Rather than depending only on isolated QSAR models or structural heuristics, the workflow shows how predictive modeling and fragment-guided screening can be used together to explore selective chemical space efficiently. The pipeline is modular, scalable and transferable, and it can be adapted to other targets or multi-isoform targets in which selectivity remains a major obstacle to therapeutic development. Collectively, the insights from this work should inform future efforts to discover novel EGFR^L858R/T790M/C797S^ inhibitors as potential therapeutic agents.

## Figures and Tables

**Table 1:** tenfold cross-validation metrics for optimized XGBoost regression models for mutant EGFR inhibitors and wild-type EGFR inhibitors.

Models	Training *R*^2^	Training *MSE*	Training *RMSE*	Training *MAE*	Test *R^2^*	Test *MSE*	Test *RMSE*	Test *MAE*
Mutant	0.74	0.28	0.53	0.40	0.75	0.29	0.54	0.39
Wild-type	0.80	0.31	0.55	0.42	0.81	0.31	0.56	0.42

**Table 2:** Hydrogen bond interaction between the hit compounds and active site residues of mutant EGFR.

Complex	Acceptor	Donor	Frac	AvgDist	AvgAng
zinc563082375	Lig@O39	SER797@NH	0.77	2.88	158.75
	Lig@O10	MET793@NH	0.11	2.90	152.99
	Lig@O39	SER797@OH	0.10	2.76	160.92
zinc1332126659	Lig@O23	MET793@NH	0.77	2.86	157.12
	Lig@O9	SER797@NH	0.63	2.86	146.61
	MET793@O	Lig@NH	0.71	2.85	160.99
zinc1600303988	Lig@O36	SER797@NH	0.59	2.91	157.32
	ASP800@OD2	Lig@OH	0.63	2.65	155.61
	ASP800@OD1	Lig@OH	0.29	2.64	155.08

**Table 3:** Binding energy (MM-PBSA) of mutant and wild-type EGFR-ligand complexes and their components (kJ/mol).

	Mutant EGFR			Wild-Type EGFR		
	zinc563082375	zinc1332126659	zinc1600303988	zinc563082375	zinc1332126659	zinc1600303988
*E*_vdw_	−29.0 ± 2.6	−33.7 ± 2.6	−35.2 ± 2.8	−28.4 ± 2.9	−31.5 ± 2.7	−30.1 ± 2.6
*E*_ele_	−9.7 ± 4.4	−20.3 ± 3.2	−22.5 ± 5.4	−14.7 ± 3.7	−10.6 ± 6.0	−14.5 ± 4.4
*E*_GB_	17.6 ± 4.2	29.9 ± 3.6	26.6 ± 4.0	22.6 ± 3.0	27.9 ± 5.5	25.3 ± 4.1
*E*_surf_	−4.2 ± 0.4	−4.9 ± 0.3	−5.3 ± 0.2	−3.6 ± 0.2	−4.6 ± 0.4	−4.5 ± 0.3
Δ*G*_gas_	−38.7 ± 6.0	−54.0 ± 3.9	−57.7 ± 4.6	−43.1 ± 3.4	−42.0 ± 6.9	−44.5 ± 4.7
Δ*G*_sol_	13.4 ± 4.1	25.0 ± 3.5	21.3 ± 4.1	19.0 ± 3.0	23.3 ± 5.4	20.8 ± 4.0
Δ*G*_binding_	−25.3 ± 3.9	−28.9 ± 3.1	−36.4 ± 3.1	−24.1 ± 3.6	−18.8 ± 3.3	−23.8 ± 3.4

## Data Availability

The original contributions presented in this study are included in the article/Supplementary Material. Further inquiries can be directed to the corresponding author.

## Supplementary Materials

The following supporting information can be downloaded at https://www.mdpi.com/article/10.3390/ijms27219796/s1.
