# Supplementary material for: ML-QSAR and Fragment Selectivity for the Identification of Fourth-Generation EGFRL858R/T790M/C797S Inhibitors
Source: Int J Mol Sci. 2026 Oct 31;27(21):9796. doi: 10.3390/ijms27219796 (PMC13763186; doi:10.3390/ijms27219796)
Supplement: Supplementary file 1 [file ijms-27-09796-s001.zip › Supplementary Figures.pdf]

ML-QSAR and Fragment Selectivity for the Identification of Fourth-Generation EGFR$^{L858R/T790M/C797S}$ Inhibitors

GuoGang Tu

Jiangxi Province Key Laboratory of Drug Target Discovery and Validation, School of Pharmaceutical Science, Jiangxi Medical College, Nanchang University, Nanchang, China

Correspondence: GuoGang Tu, tugg110@163.com

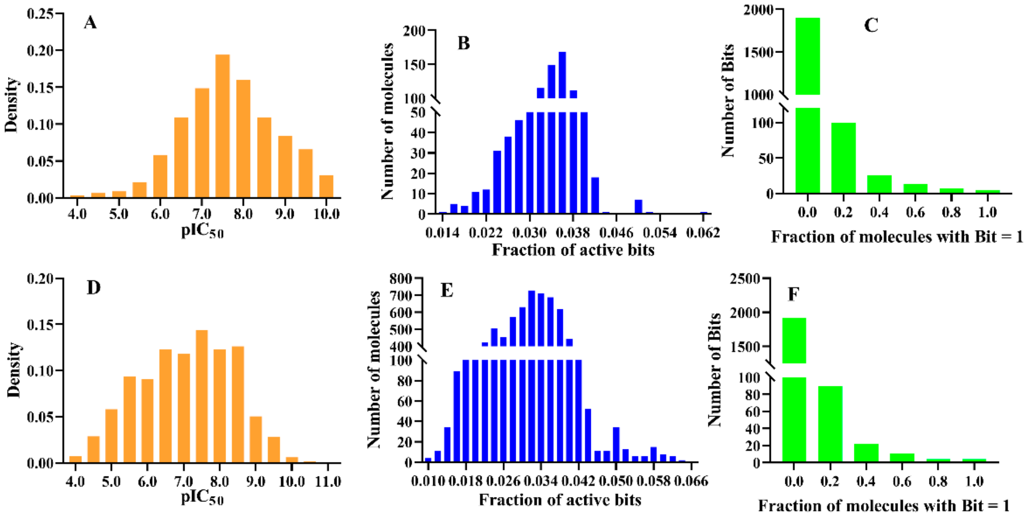

Figure S1. pIC_{50} distribution, ECFP4 sparsity, and fingerprint activation frequency (pre-refinement). A-C: mutant EGFR inhibitors, D-F: wild-type EGFR inhibitors, Panels show (A, D) pIC_{50} distributions, (B, E) ECFP4 sparsity, and (C, F) fingerprint activation frequency profiles for each group inhibitors.

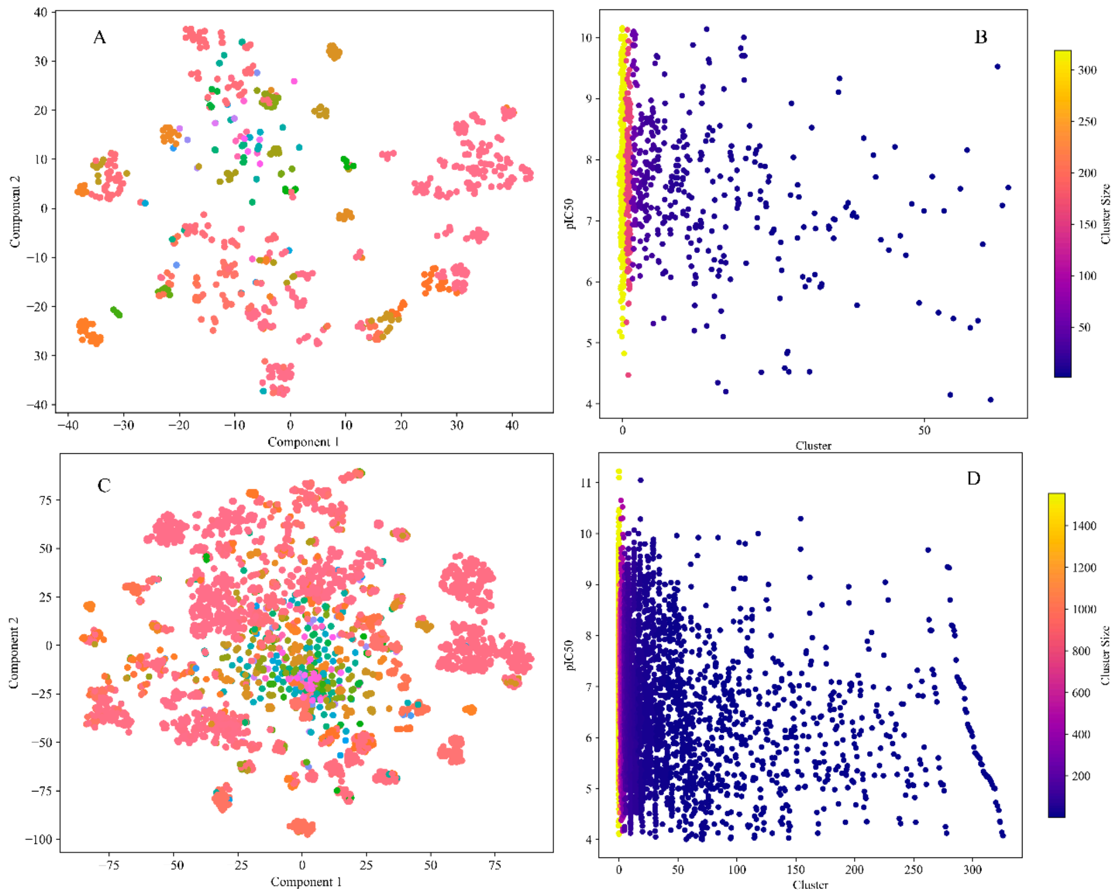

Figure S2. Clustering analysis of EGFR inhibitors (pre-refinement) based on ECFP4 Tanimoto similarity (threshold = 0.3) and t-SNE dimensionality reduction. (A-B) mutant EGFR inhibitors, (C-D) wild-type EGFR inhibitors. Panels (A, C) present t-SNE projections of Butina clusters colored by cluster identity, highlighting the chemical diversity and spatial separation of compounds in two dimensions. Panels (B, D) show cluster size distributions colored by potency and corresponding mean pIC_{50} values, illustrating the variability in activity across structural groups.

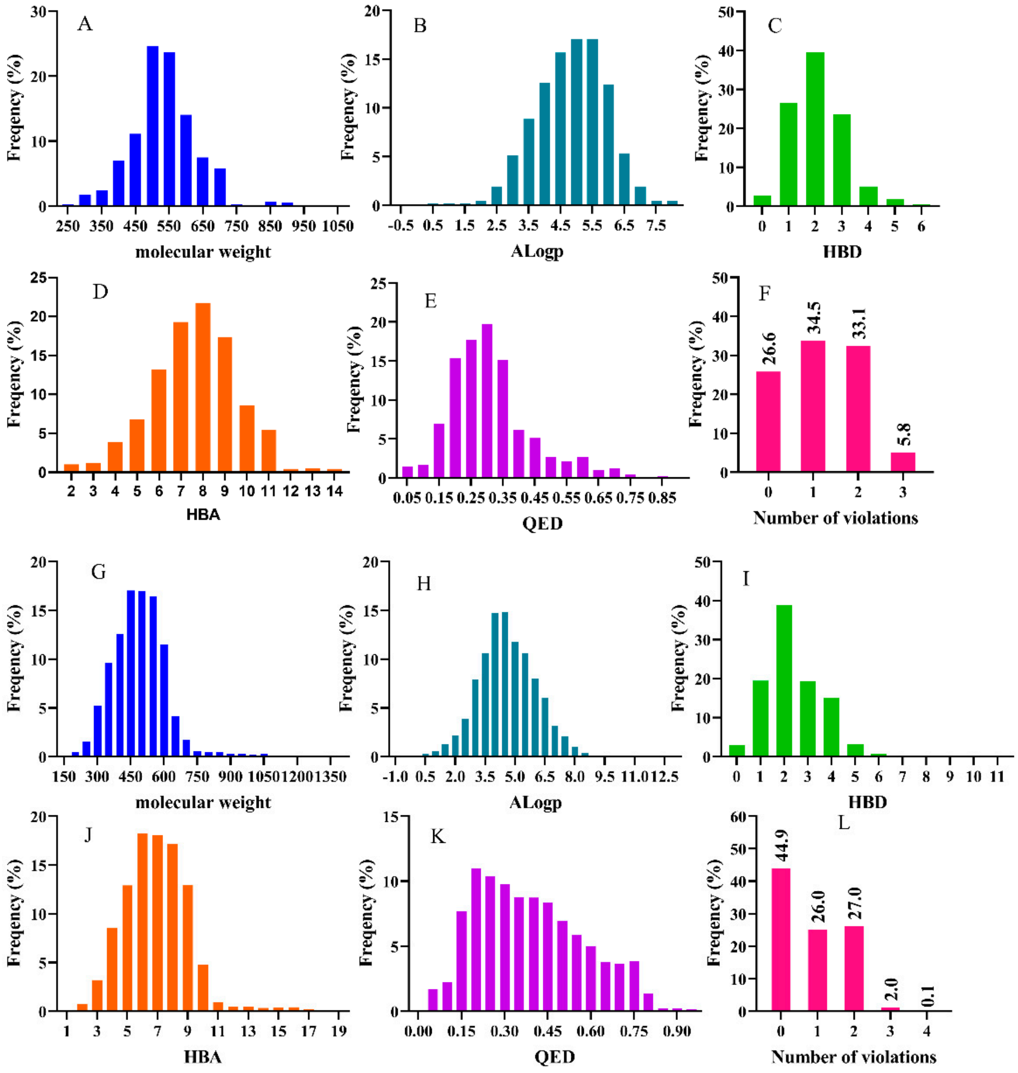

Figure S3. Physicochemical property and drug-likeness profiles of EGFR inhibitors (pre-refinement). (A-F) mutant EGFR inhibitors, (G-L) wild-type EGFR inhibitors. Panels present the distributions of key molecular properties: (A, G) molecular weight, (B, H) ALogP, (C, I) hydrogen-bond donors (HBD), (D, J) hydrogen-bond acceptors (HBA), (E, K) quantitative estimate of drug-likeness (QED), and (F, L) number of Lipinski's Rule of Five violations.

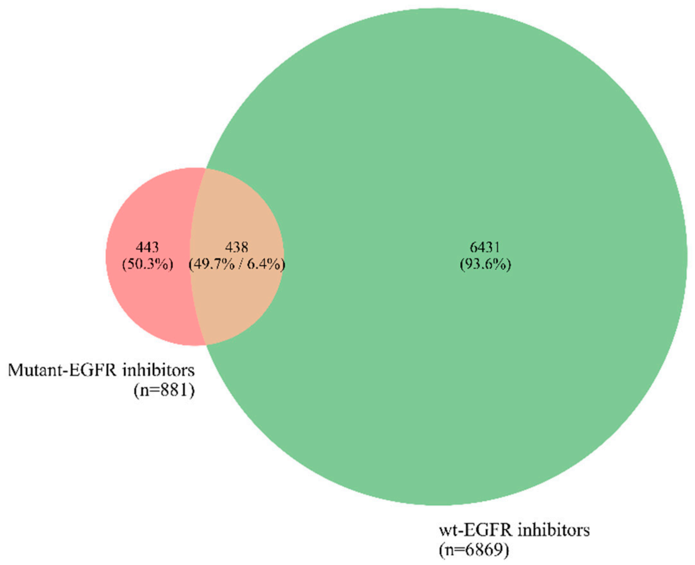

Figure S4. Venn diagram illustrating the overlap of compounds (pre-refinement)

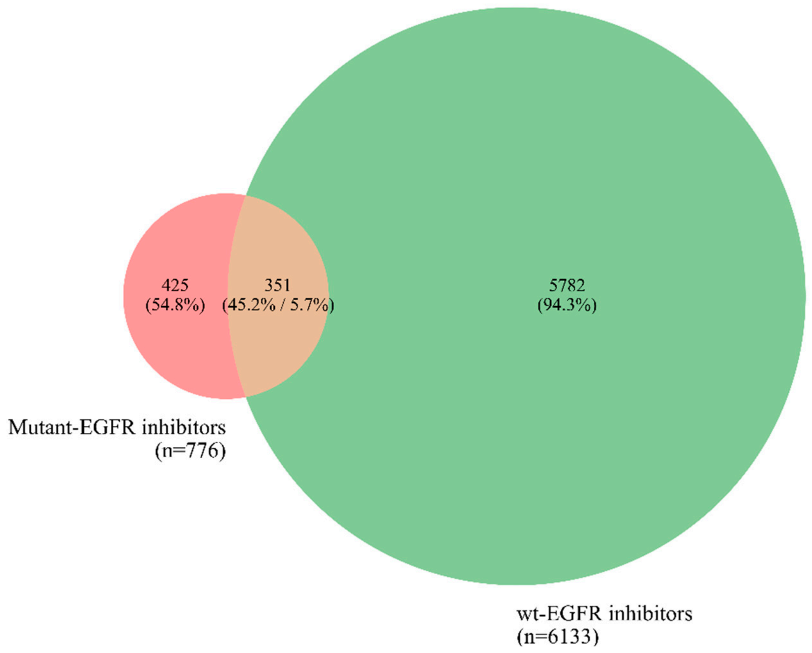

Figure S5. Venn diagram illustrating the overlap of compounds (post-refinement)

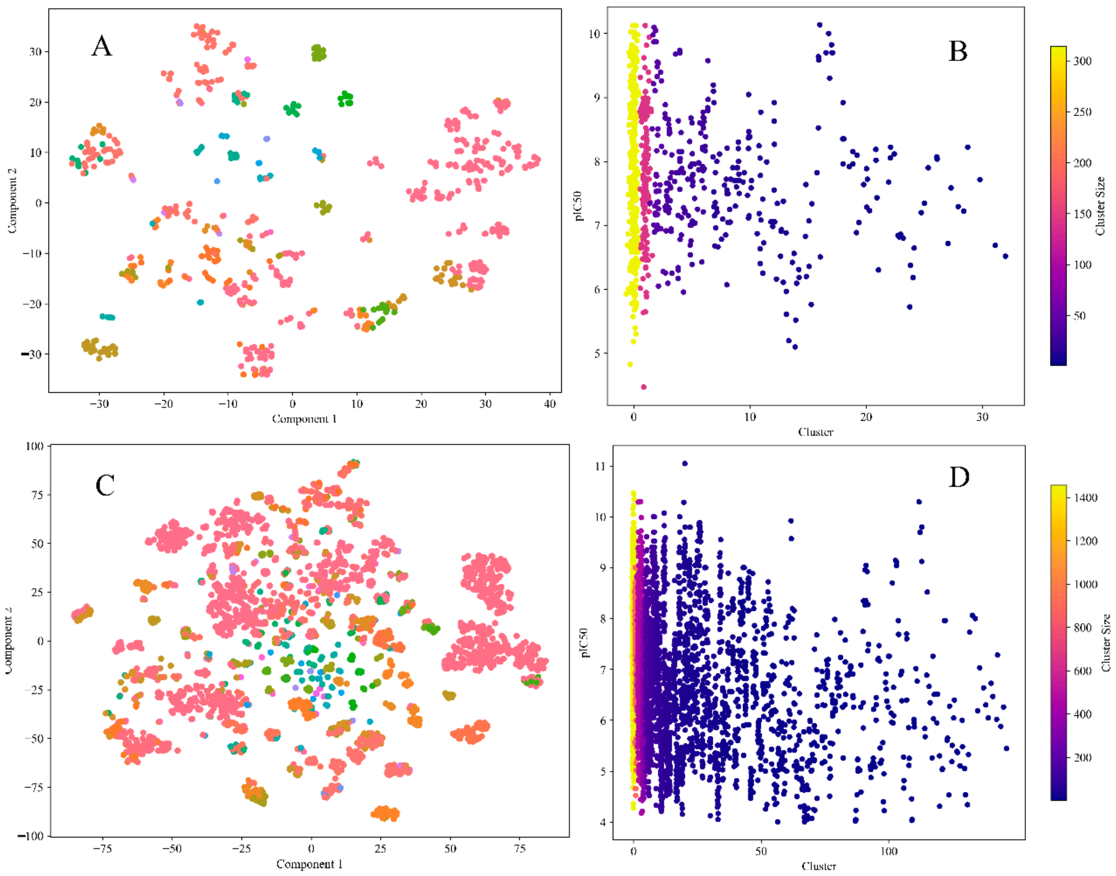

Figure S6. Post-refinement clustering of EGFR inhibitors using ECFP4 fingerprints and t-SNE dimensionality reduction. (A-B) mutant EGFR inhibitors, (C-D) wild-type EGFR inhibitors. Panels (A, C) display t-SNE projections of Butina clusters, demonstrating cohesive and chemically consistent compound groupings. Panels (B, D) show cluster size distributions colored by potency and corresponding mean pIC_{50} values.

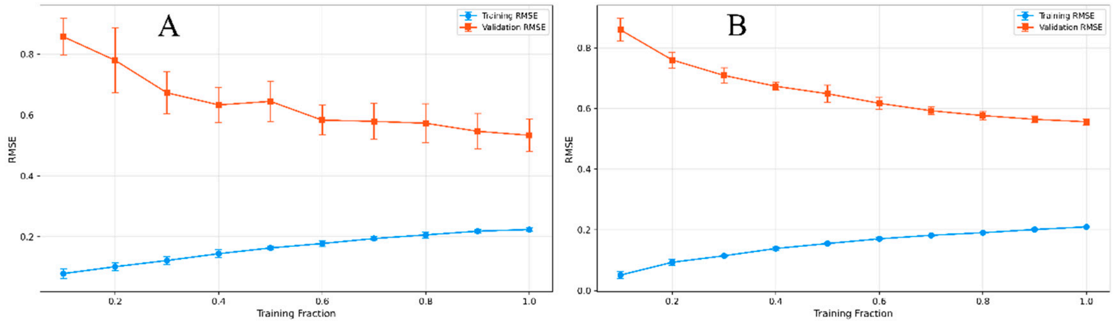

Figure S7. Learning curves for XGBoost regression models of (A) mutant EGFR inhibitors, (B) wild-type EGFR inhibitors, illustrating near-synchronous convergence of training and validation RMSE.

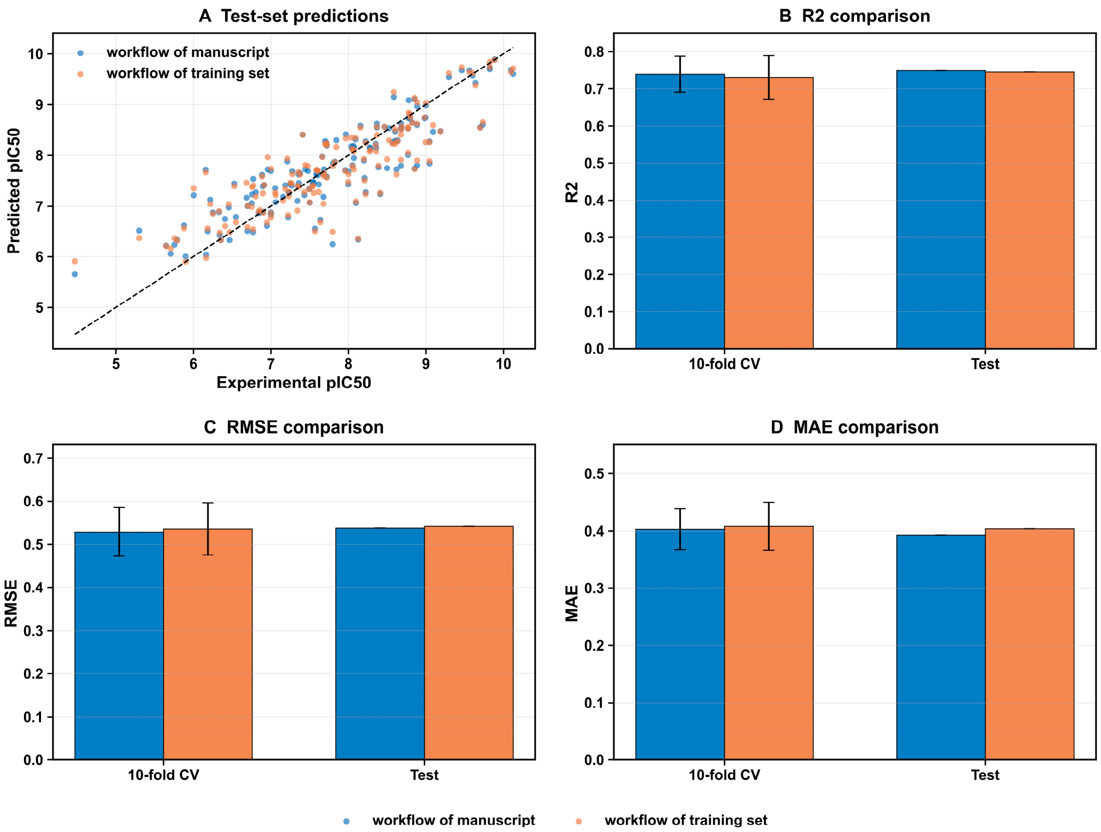

Figure S8. Performance comparison of the mutant EGFR QSAR models under the training+test set and training-set-only feature-selection workflows. (A) Experimental versus predicted pIC50 for the test set (n = 1,230). Blue and orange indicate the full-data workflow and the training-set-only workflow, respectively. (B–D) R^2, RMSE, and MAE for 10-fold cross-validation and the test set. Error bars indicate standard deviations across the 10 CV folds. Under this procedure, 298 features were retained, compared with 296 in the full-data selection. The obtained results were $R^2 = 0.745$, RMSE = 0.542, and MAE = 0.404, compared with $R^2 = 0.739$, RMSE = 0.529, and MAE = 0.403 for the full-data workflow. Paired bootstrap analysis showed that these differences were small and not statistically distinguishable.

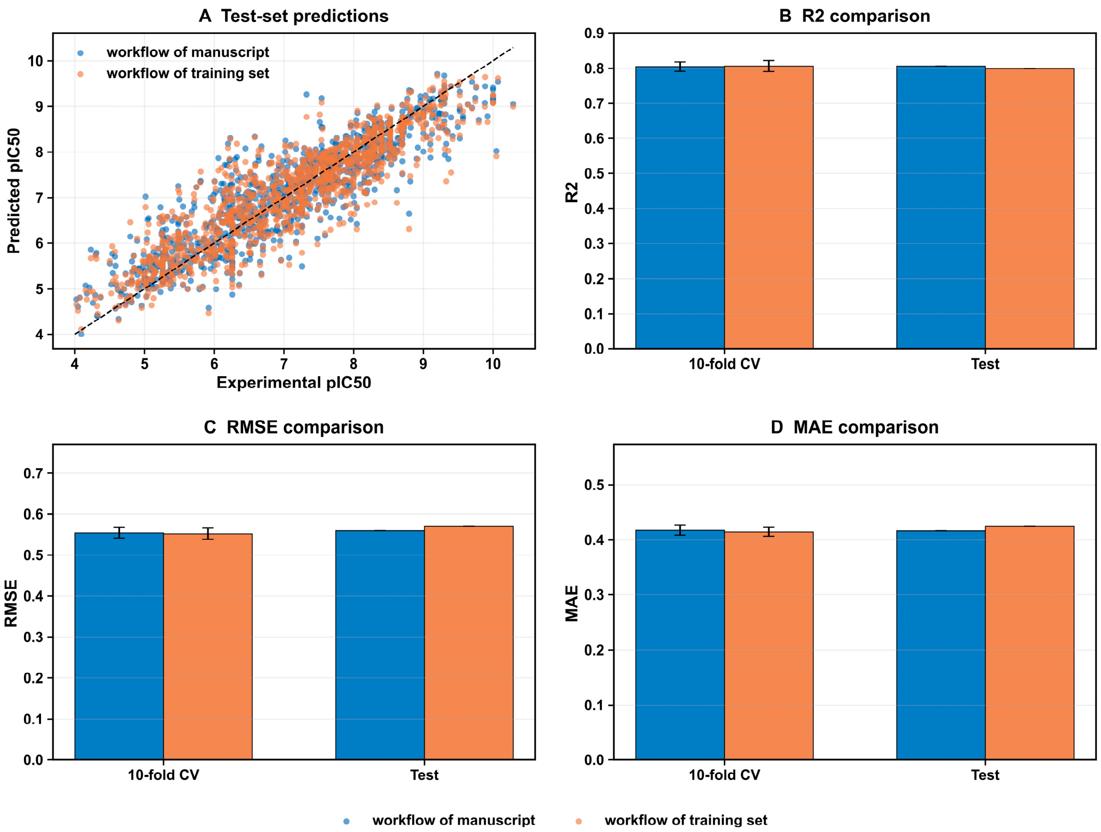

Figure S9. Performance comparison of the wild-type EGFR QSAR models under the training+test and training-set-only feature-selection workflows. (A) Experimental versus predicted pIC50 for the test set (n = 1,230). Blue and orange indicate the full-data workflow and the training-set-only workflow, respectively. (B–D) R^2, RMSE, and MAE for 10-fold cross-validation and the test set. Error bars indicate standard deviations across the 10 CV folds. The results show that 306 features were retained, compared with 301 in the full-data selection. The obtained results were $R^2 = 0.807$, RMSE = 0.553, and MAE = 0.305, compared with $R^2 = 0.807$, RMSE = 0.560, and MAE = 0.308 for the full-data workflow. Paired bootstrap analysis showed that these differences were small and also not statistically distinguishable.

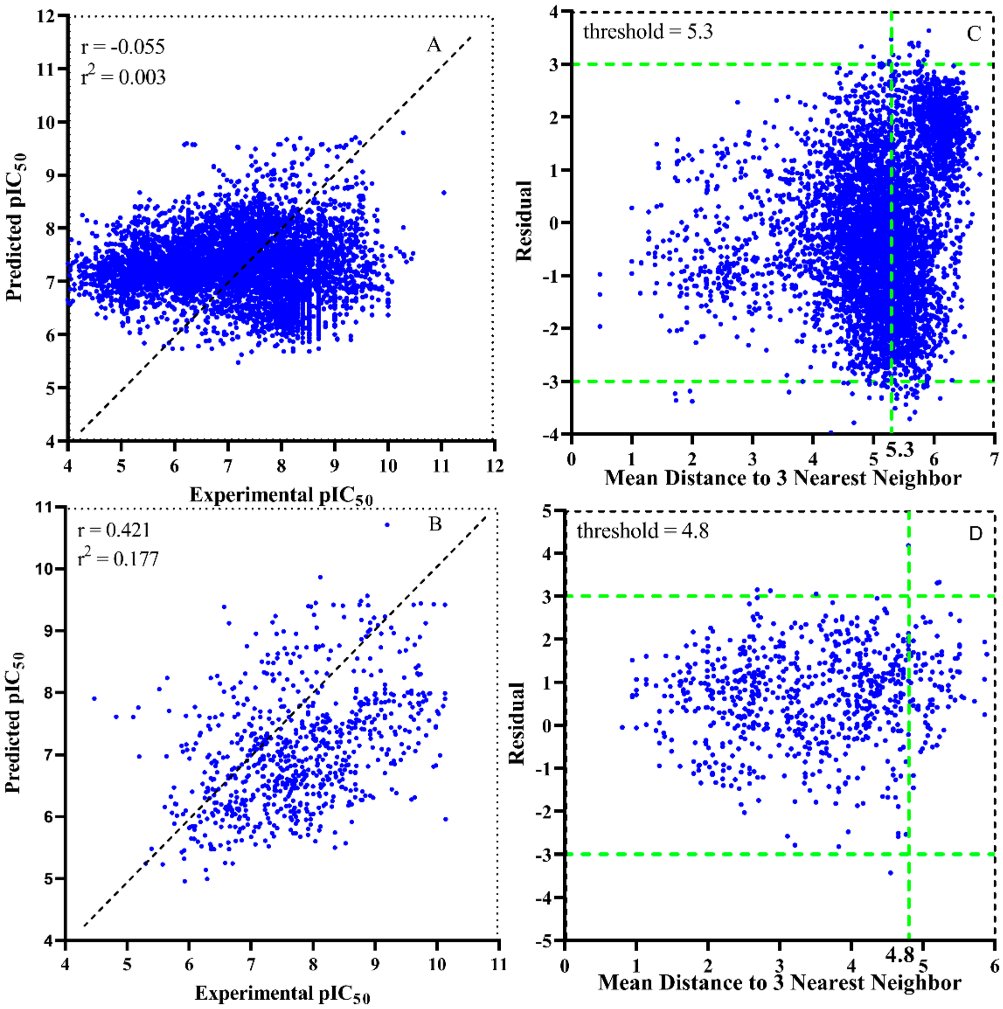

Figure S10. Cross-dataset prediction performance. (A, B) experimental vs predicted pIC_{50} values, applicability domain plot for mutant model vs WT-EGFR inhibitors, (C, D) experimental vs predicted pIC_{50} values, applicability domain plot for WT model vs mutant-EGFR inhibitors

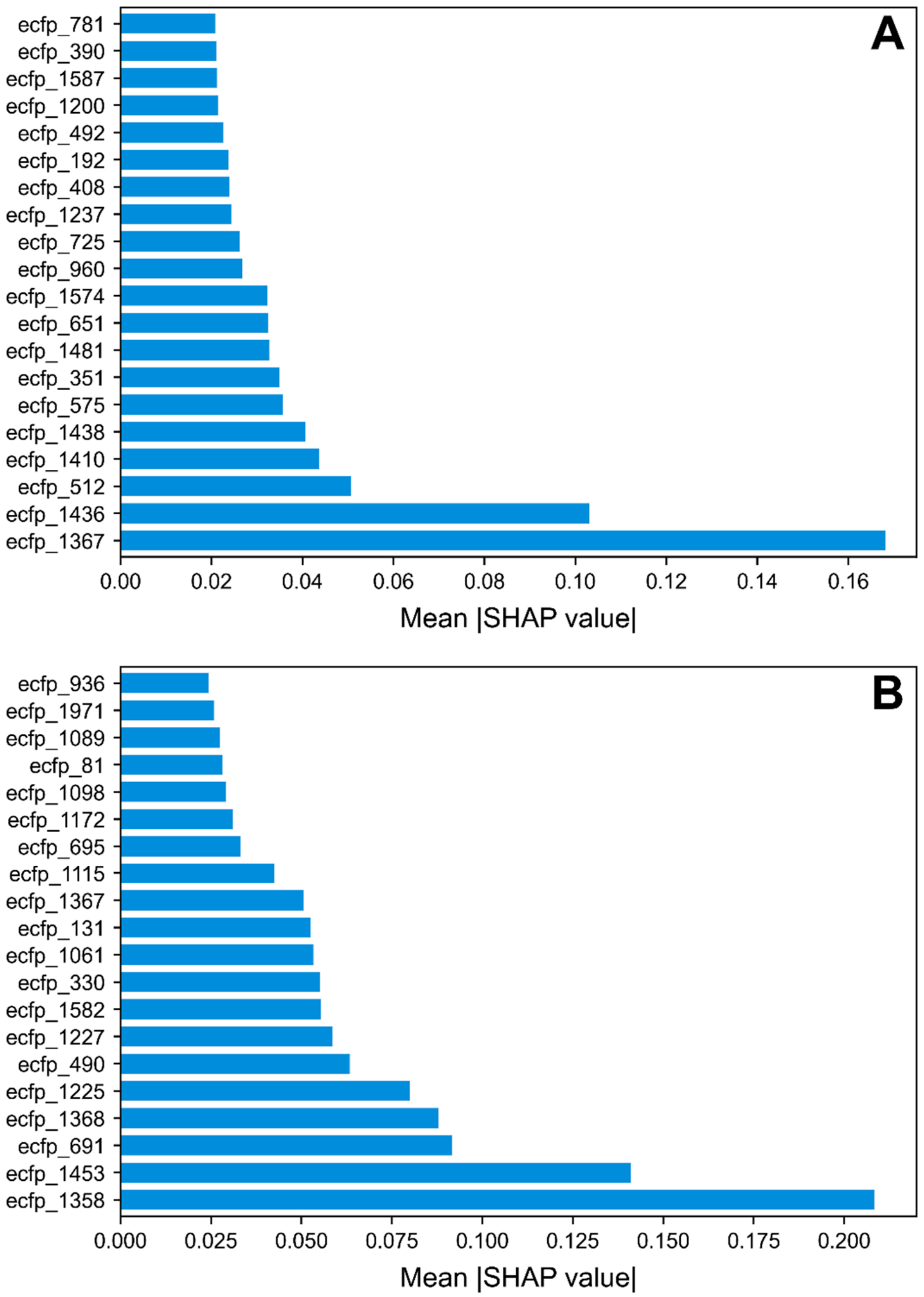

Figure S11. SHAP feature importance plots. Each panel (A, B) corresponds to mutant model and WT model respectively, illustrating the mean absolute SHAP values of the top 20 most influential ECFP4 features. Higher mean SHAP values indicate stronger contributions of the corresponding molecular substructures to the model's predictive performance. These feature importance profiles highlight the distinct and shared structural determinants governing EGFR inhibitions.

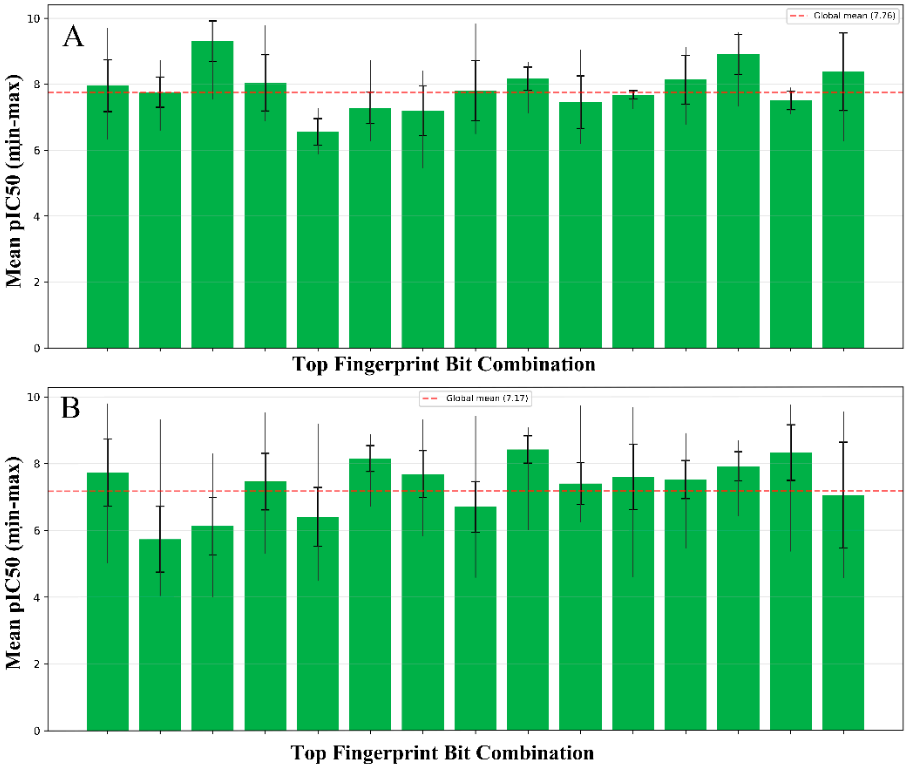

Figure S12. Experimental activity trends confirm additive effects of SHAP-identified fingerprints. Each panel (A, B) corresponds to mutant and WT model. Compounds were grouped based on the number of top ten SHAP-identified ECFP4 fingerprint. The y-axis shows the mean experimental pIC_{50}, and the error bars indicate the observed activity range. The increase in mean activity with a higher count of influential bits supports the additive contribution of key substructural features to inhibitors selectivity.

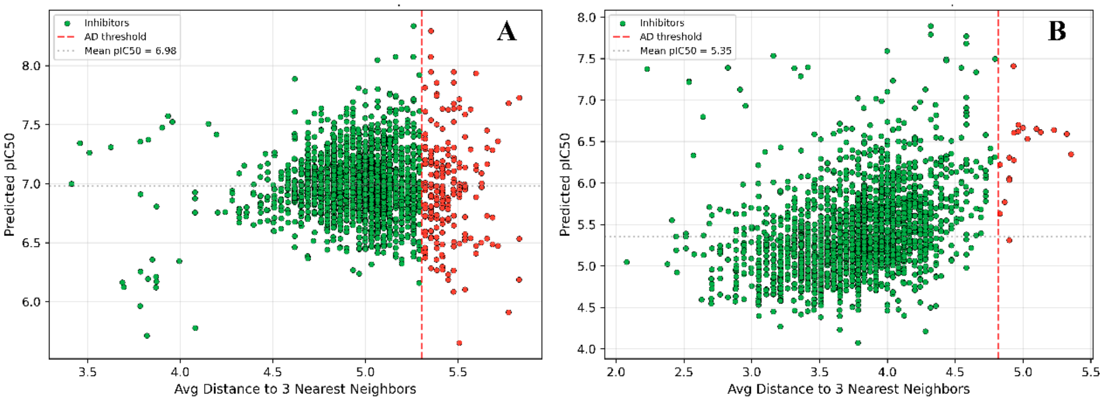

Figure S13. Applicability domain plot for bioactive fragment analysis by (A) mutant QSAR and (B)WT-QSAR

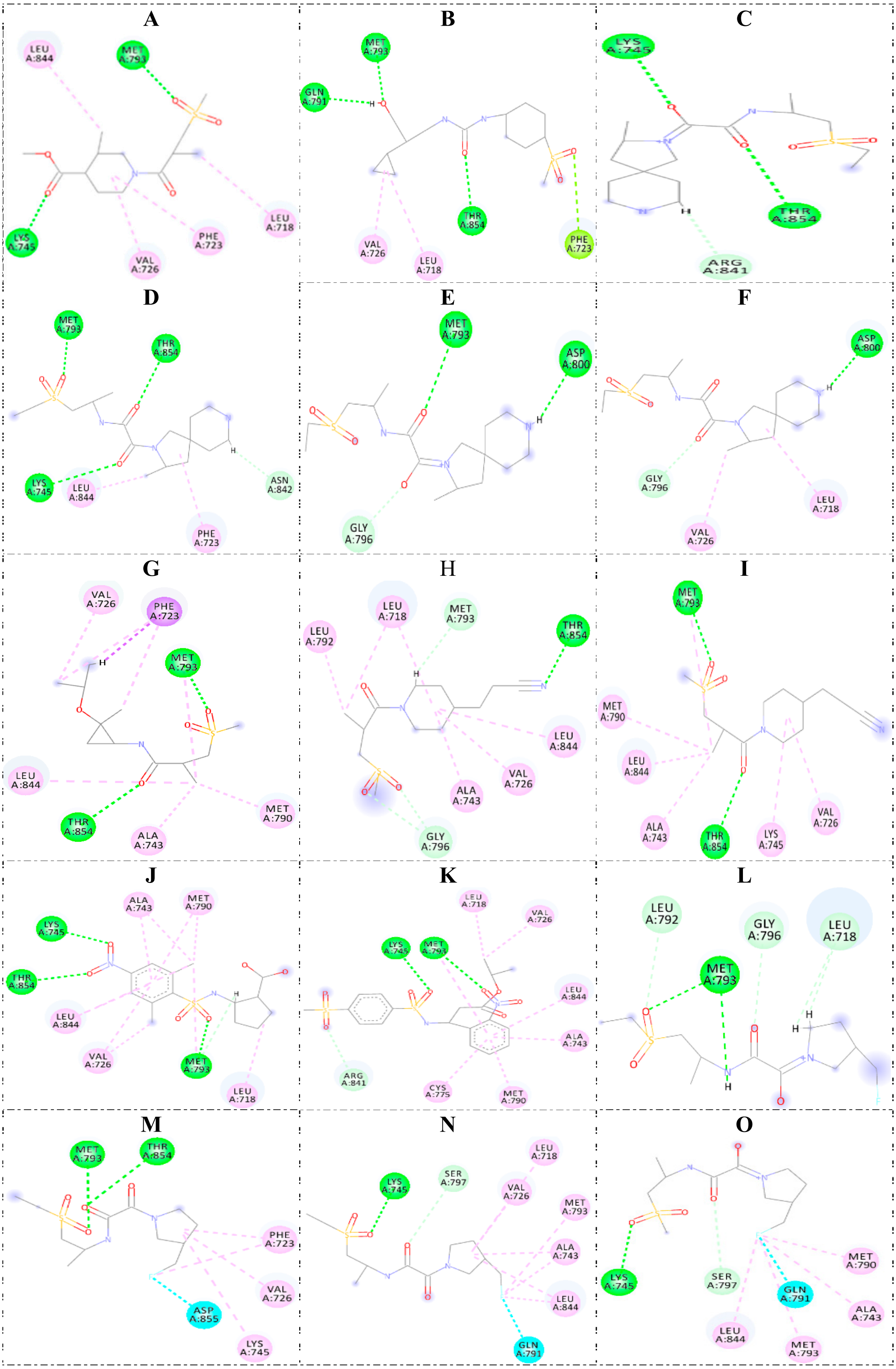

A
LEU A:844
MET A:793
LYS A:745
VAL A:726
PHE A:723
LEU A:718
B
MET A:793
GLN A:791
THR A:854
VAL A:726
LEU A:718
PHE A:723
C
LYS A:745
THR A:854
ARG A:841
D
MET A:793
THR A:854
LYS A:745
LEU A:844
ASN A:842
PHE A:723
E
MET A:793
ASP A:800
GLY A:796
F
ASP A:800
GLY A:796
LEU A:718
VAL A:726
G
VAL A:726
PHE A:723
MET A:793
LEU A:844
THR A:854
ALA A:743
MET A:790
H
LEU A:718
MET A:793
LEU A:792
THR A:854
LEU A:844
VAL A:726
ALA A:743
GLY A:796
I
MET A:793
MET A:790
LEU A:844
ALA A:743
THR A:854
LYS A:745
VAL A:726
J
ALA A:743
MET A:790
LYS A:745
THR A:854
LEU A:844
VAL A:726
MET A:793
LEU A:718
K
LEU A:718
VAL A:726
LYS A:745
MET A:793
LEU A:844
ALA A:743
ARG A:841
CYS A:775
MET A:790
L
LEU A:792
GLY A:796
LEU A:718
MET A:793
M
MET A:793
THR A:854
PHE A:723
VAL A:726
ASP A:855
LYS A:745
N
LYS A:745
SER A:797
LEU A:718
VAL A:726
MET A:793
ALA A:743
LEU A:844
GLN A:791
O
LYS A:745
SER A:797
MET A:790
GLN A:791
ALA A:743
LEU A:844
MET A:793

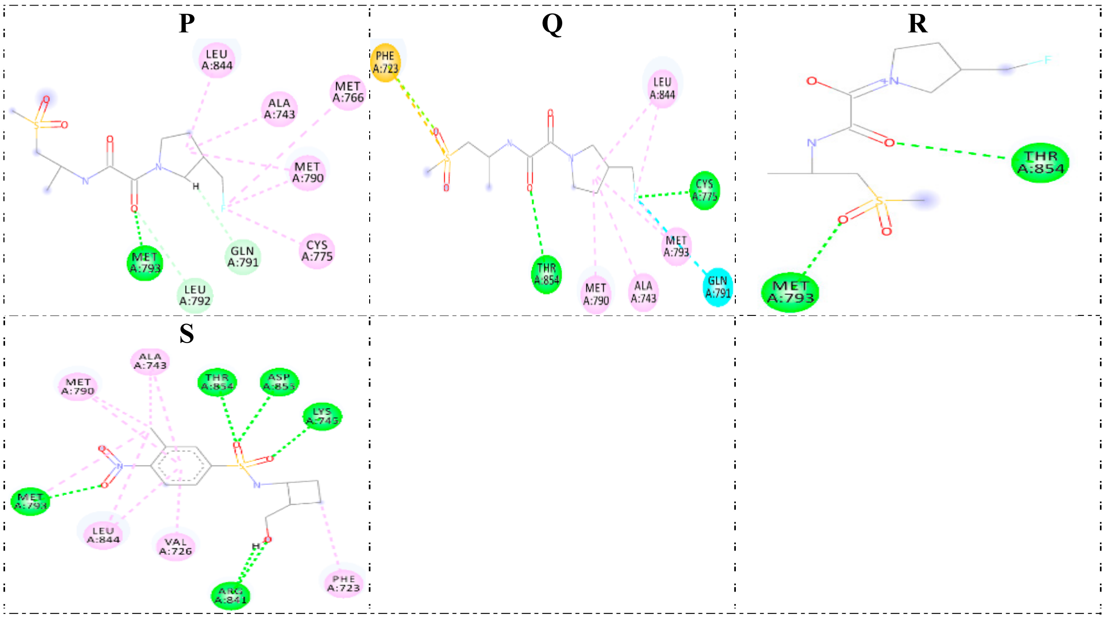

Figure S14. The docking analysis of A (zinc356832345), B (zinc358949772), C (zinc424550790), D (zinc424550792), E (zinc424550794), F (zinc424550796), G (zinc529966979), H (zinc578896256), I (zinc578896257), J (zinc592152114), K (zinc602078062), L (zinc1332126656), M (zinc1332126657), N (zinc1332126658), O (zinc1334503970), P (zinc1334503971), Q (zinc1334503972), R (zinc1334503974), S (zinc1607708271) with the active site of EGFR$^{L858R/T790M/C797S}$. Hydrogen bonds are indicated by green dashed lines. The results indicate that no hydrogen bond was formed between the ligands and the mutant ser797.

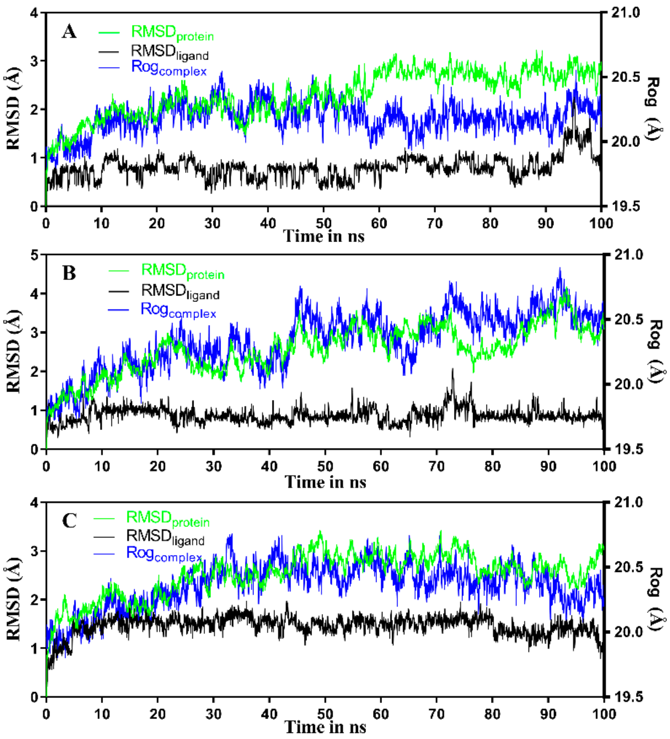

Figure S15. RMSD and RoG plots for wild-type EGFR-ligand complex. A: EGFR-zinc563082375 complex, B: EGFR-zinc1332126659 complex, C: EGFR-zinc1600303988 complex. The results indicate that all protein–ligand complexes are conformationally stable in the dynamic conditions investigated.

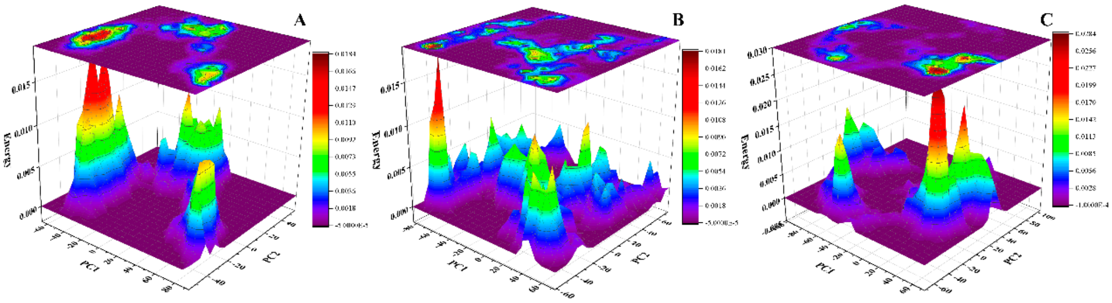

Figure S16. FEL plots for wild-type EGFR-ligand complex. A: EGFR-zinc563082375 complex, B: EGFR-zinc1332126659 complex, C: EGFR-zinc1600303988 complex. The deep purple areas represent the lowest energy conformers, while the deep red areas signify the highest energy conformers. The abundance of purple regions indicates that the conformations are thermodynamically favorable and stabilize the complex in an MD simulation.
